# CRISPR/Cas9-based repair of a heterozygous HNF1A mutation in patient-derived hiPSCs

**DOI:** 10.1007/s00439-026-02863-0

**Published:** 2026-08-12

**Authors:** Dawid Skoczek, Jerzy Hohendorff, Maciej T. Malecki, Alicia Roig-Merino, Rasmus O. Bak, Neli Kachamakova-Trojanowska

**Affiliations:** 1 Malopolska Centre of Biotechnology, Jagiellonian University, Gronostajowa str 7a, 30-387 Krakow, Poland; 2 Doctoral School of Exact and Natural Sciences, Jagiellonian University, Lojasiewicza str 11, 30-387 Krakow, Poland; 3 Department of Metabolic Diseases, Jagiellonian University Medical College, Jakubowskiego str 2, 30-688 Krakow, Poland; 4 MaxCyte Inc, Rockville, MD 20850 USA; 5 Department of Biomedicine, Aarhus University, Aarhus, 8000 Denmark

## Abstract

Human induced pluripotent stem cells (hiPSCs) represent a powerful platform for disease modeling, especially in monogenic diseases as they preserve the donor’s genetic background while enabling directed differentiation into disease-relevant cell types. This makes them highly suitable for studying disease mechanisms in a patient-specific and physiologically relevant context. Although CRISPR/Cas9 is widely applied for genome editing, precise correction of pathogenic variants in hiPSCs remains challenging due to the lack of standardized CRISPR component selection and experimental design. Here, we describe an optimized CRISPR-based strategy for correcting a heterozygous *HNF1A* frameshift mutation (c.235_236insG; p.Glu79Glyfs*16) in HNF1A-MODY patient-derived hiPSCs. Using electroporation, we efficiently delivered CRISPR components, including a ribonucleoprotein complex of Cas9 and single-guide RNA, along with a single-stranded oligodeoxynucleotide repair template. Corrected hiPSC lines were validated for pluripotency, absence of exogenous reprogramming factors, and off-target effects. Additionally, we discuss key technical challenges encountered during the editing process and provide practical recommendations that may improve the generation of mutation-corrected hiPSC lines. These guidelines could serve as a useful reference for researchers employing CRISPR-based strategies for generation of reliable disease modelling tools.

**Supplementary Information:**

The online version contains supplementary material available at 10.1007/s00439-026-02863-0.

## Introduction

Maturity-onset diabetes of the young (MODY) is a hereditary monogenic form of diabetes accounting for 1–5% of all diabetes cases, though its true prevalence is likely underestimated due to frequent misdiagnosis with type 1 (T1D) or type 2 diabetes (T2D), leading to inappropriate treatment strategies (Skoczek et al. 2021). HNF1A-MODY is the most common MODY subtype, with 30–50% of all MODY cases. It is caused by mutations in the *HNF1A* gene, most of which are heterozygous. Hepatocyte nuclear factor-1 alpha (HNF1A) is a transcription factor essential for pancreatic beta-cell development, insulin secretion, and glucose homeostasis (Valkovicova et al. 2019). While individuals with HNF1A-MODY often exhibit milder hyperglycemia than those with T1D or T2D, they are at high risk for vascular complications such as retinopathy and nephropathy (Delvecchio et al. 2020).

The *HNF1A* gene, located on chromosome 12q24.31, consists of 10 exons and undergoes alternative splicing, producing multiple transcript variants with distinct isoforms. To date, 1126 mutations associated with HNF1A-MODY have been identified, with approximately 70% classified as pathogenic, likely pathogenic or with uncertain significance. Within these mutations majority are missense (56%), followed by frameshift (15%) and nonsense (8%) mutations (Bellanné-Chantelot et al. 2008). Even though the mutations are distributed throughout the gene, the recurrent c.872dupC; p.Gly292ArgfsTer25 (ClinVar Variation ID: 14927) frameshift mutation, which truncates the transactivation domain, has been reported in several studies as the most common variant associated with HNF1A-MODY (Yamagata et al. 1998; Colclough et al. 2013).

Human induced pluripotent stem cells (hiPSCs) have emerged as a groundbreaking tool for disease modeling, particularly for monogenic disorders such as HNF1A-MODY. Unlike traditional cell lines or animal models, hiPSCs preserve the donor’s genetic background while maintaining the ability to differentiate into disease-relevant cell types, making them highly suitable for studying disease mechanisms in a patient-specific and physiologically relevant context (Doss and Sachinidis 2019). Furthermore, hiPSCs provide an unlimited, renewable source of patient-derived cells while circumventing ethical concerns associated with embryonic stem cells (Moradi et al. 2019). hiPSCs have been successfully used to model a wide range of monogenic disorders, including cystic fibrosis (MIM #219700), caused by pathogenic variants in *CFTR* (Berical et al. 2022), Duchenne muscular dystrophy (MIM #310200), resulting from variants in *DMD* (Lin et al. 2015), and sickle cell disease (MIM # 603903) caused by pathogenic variants in *HBB* (Ou et al. 2016). In the context of monogenic metabolic disorders, hiPSC-based models have provided valuable insights into diseases such as HNF1A-MODY (MIM #600496) (Kachamakova-Trojanowska et al. 2019), Wolfram syndrome (MIM #222300), caused by pathogenic variants in *WFS1*(Ahuja et al. 2024), and familial hypercholesterolemia (MIM #143890), resulting from pathogenic variants in *LDLR* (Okada et al. 2019). These models have become essential tools for investigating disease mechanisms, evaluating therapeutic strategies, and validating gene-editing approaches.

A key advantage of isogenic hiPSC lines in disease modelling is their ability to minimize genetic variability, which is crucial for accurately studying the effects of specific pathogenic variants. These lines differ only in a single, precisely introduced genetic alteration, such as a pathogenic variant correction or insertion, allowing isolating the direct impact of the genetic change under investigation. This approach eliminates confounding factors, such as natural genetic variation between individuals, which can obscure the effects of a particular variant and complicate phenotype interpretation. To generate such isogenic lines, CRISPR/Cas9 genome editing is currently widely used, enabling precise correction of pathogenic variants in patient-derived hiPSCs while maintaining the genetic background of the individual. Additionally, CRISPR/Cas9 can be alternatively used to introduce disease-relevant variants into unaffected donor-derived hiPSCs, expanding the repertoire of available models for studying monogenic disorders and facilitating drug development and therapeutic testing (Volpato and Webber 2020; Cerneckis et al. 2024).

Despite the advantages of CRISPR/Cas9 in genome editing, its application in hiPSC-based isogenic models is hindered by several technical challenges. While introducing mutations via non-homologous end joining (NHEJ) is relatively efficient, this repair pathway is error-prone and often results in a spectrum of small insertions or deletions (indels), making it unsuitable for precise variant insertion or repair (Miyaoka et al. 2016). A precise mutation correction would require homology-directed repair (HDR), which is a less efficient repair pathway with an estimated efficiency of 1–10% in hiPSCs, with variations depending on the targeted genomic locus (Byrne et al. 2014; Maguire et al. 2019; Simkin et al. 2022).

HDR enables the correction of patient-specific variants or the introduction of a defined sequence change. However, it requires the presence of a repair template (also called donor DNA), typically in the form of a single-stranded oligodeoxynucleotide (ssODN) or a plasmid to guide accurate genomic modification (Liao et al. 2024). Without this template, the cell will predominantly rely on the error-prone NHEJ pathway, where the likelihood of accurate gene editing is significantly reduced or not possible at all. Still, in the presence of a repair template, the NHEJ and HDR pathways compete, which is reflected in the allelic outcomes that may on a cellular level be a mix of indels, precise gene editing, or no edit at all. Off-target effects, clonal variability, and limitations in editing efficiency further complicate the generation of precise disease models. These technical hurdles become even more pronounced when considering therapeutic applications. Achieving efficient, precise gene correction for clinical use is far more complex due to additional factors such as delivery challenges, immune responses, and the need for long-term safety and efficacy validation (Taha et al. 2022).

In the current study, we show the challenges that can appear when generating an isogenic control hiPSC lines using CRISPR/Cas9-mediated gene correction and how these challenges could be tackled. To create an isogenic disease model for HNF1A-MODY, we performed correction of a heterozygous *HNF1A* frameshift mutation (c.235_236insG; p.Glu79Glyfs*16) present in patient-derived hiPSCs. Using electroporation, we introduced a ribonucleoprotein (RNP) complex composed of Cas9 and single-guide RNA (sgRNA), along with a single-stranded ssODN repair template to facilitate HDR-mediated correction. Even though we applied all known strategies for increased HDR efficiency, the efficiency of the CRISPR-based mutation repair remained limited. Our study highlights key areas for optimization, including experimental workflow refinement, sgRNA design, clonal handling, and the selection of successfully repaired clones. Through the targeted correction of a disease associated *HNF1A* variant, we demonstrate how these variables impact editing outcomes and propose strategies to improve the reproducibility and reliability of CRISPR-mediated gene correction in disease modeling applications.

## Materials and methods

### Generation of hiPSC lines

A patient-specific hiPSCs line was generated from peripheral blood mononuclear cells (PBMCs) following blood collection, which was conducted after obtaining the informed consent of the patient and the approval of the Jagiellonian University Bioethical Committee (No. 1072.6120.310.2018). PBMCs were reprogrammed using non-integrating Sendai vectors (CytoTune-iPS 2.0 Sendai Reprogramming Kit, Thermo Fisher Scientific) according to the manufacturer’s protocol for a feeder-free system. After approximately three weeks, emerging hiPSCs colonies were manually selected and expanded. The successfully derived clone was verified for pluripotency markers expression and spontaneous differentiation into the three germ layers.

### hiPSCs maintenance

hiPSCs were cultured on Geltrex-coated (Gibco) wells in Essential 8 Medium (E8, Gibco) at 37 °C in a humidified incubator at 5% CO_2_ (standard conditions). For subculturing, the hiPSCs were detached with 0.5 mM EDTA in PBS w/o Ca^2+^ and Mg^2+^. After harvesting, the cells were resuspended in a culture medium supplemented with 10 µM ROCK inhibitor (ROCK; Y-27632; Focus Biomolecules) and maintained in standard conditions. After 24 h, the medium was changed to E8 and later was exchanged daily.

### DNA isolation and PCR

Genomic DNA was extracted from cultured cells using the Genomic Mini Kit (A&A Biotechnology) according to the manufacturer’s protocol. PCR amplification was performed using KAPA2G Fast Genotyping Mix (Roche) and specific primers (Supplementary Table 1). A total of 60 ng of genomic DNA was used as a template, and the PCR conditions are detailed in Supplementary Table 2. PCR products were analysed by 2% agarose gel electrophoresis in TAE buffer at 70 V for 45 min.

### gRNA in silico design

The gRNAs were identified with an 80-nucleotide *HNF1A* sequence surrounding the mutation site (40 nt upstream and 40 nt downstream: GAGACTCGGGGCTCCGAGGACGAGACGGACGACGATGGGGAAGACTTCACGCCACCCATCCTCAAAGAGCTGGAGAACCT). Three online tools: CRISPOR (crispor.gi.ucsc.edu/crispor.py), CHOPCHOP (version 3, chopchop.cbu.uib.no) and Cas-Designer (rgenome.net/cas-designer) were employed to select high-scoring gRNAs with minimal predicted off-target activity (Concordet and Haeussler 2018; Labun et al. 2019; Park et al. 2015). Using these tools, sgRNAs were chosen for further studies (Supplementary Table 3). During sgRNA selection, a potential off-target site in the *GSE1* gene was identified for the most promising sgRNA-1 and accounted for its downstream analyses.

### gRNA cloning into a plasmid encoding CRISPR/Cas9 system

Complementary gRNA oligos were synthesized (Sigma-Aldrich) with short flanking sequences to facilitate cloning into the PX459 vector (Addgene Plasmid #62988**)**. The top oligo strand at 5’ end was extended with “CACCG”, while the bottom strand contained “AAAC” at the 5’ end and an additional “C” at the 3’ end to ensure compatibility. The expression plasmid was linearized by digestion with *Bbs*I at 37 °C for 30 min to generate compatible overhangs. The digested plasmid was then subjected to electrophoresis and purified using the Gel Out kit (A&A Biotechnology) to remove any residual fragments. Meanwhile, the oligonucleotides were phosphorylated and annealed in the presence of T4 polynucleotide kinase (PNK; New England BioLabs) and ligation buffer, incubated at 37 °C for 30 min, then heated to 95 °C for 5 min before gradually cooling to 25 °C to promote proper annealing.

To integrate the gRNA into the expression vector, the annealed oligonucleotides were diluted and ligated into the linearized plasmid using Quick Ligase (New England BioLabs) at room temperature for 60 min. The ligation product was then transformed into competent *Stbl3* bacteria (New England BioLabs) through heat shock. Cells were incubated on ice for 20 min, heat shocked at 42 °C for 45 s and returned to ice for 2 min. Following transformation, 150 µL of S.O.C. medium (Invitrogen) was added, and the culture was incubated at 37 °C for 45 min with shaking. The transformed bacteria were plated on LB agar (BioShop) supplemented with 100 µg /mL ampicillin (Sigma-Aldrich) and incubated overnight at 37 °C. The following day, the transformed bacterial colonies were selected and cultured separately in LB broth containing ampicillin at 37 °C with shaking. The next day, plasmids were extracted using the Plasmid Miniprep kit (Zymo Research) according to the manufacturer’s protocol. Successful cloning of the gRNA was confirmed by restriction enzyme analysis and Sanger sequencing.

### gRNA testing in patient-specific hiPSCs

PX459 plasmid containing Cas9 and the cloned gRNA was electroporated into patient-specific hiPSCs using Nucleofector IIb (Lonza/Amaxa Biosystems) and B0-16 program. 2.0 × 10⁶ hiPSCs were electroporated with 5 µg of plasmid DNA, containing Cas9 and selected gRNA, was mixed with the Human Stem Cell Nucleofector^®^ Kit 1 (Lonza). On the next day, successfully transfected cells were selected with puromycin (0.35 µg/mL) for 24 h. Genomic DNA was isolated 96 h post-electroporation, and the targeted locus was PCR-amplified, and analysed by Sanger sequencing. The editing efficiency of each gRNA was assessed using the TIDE (Tracking of Indels by Decomposition) and DECODR (Deconvolution of Complex DNA Repair) web-based analytical tools (Brinkman et al. 2018; Bloh et al. 2021). However, the precise evaluation of CRISPR-Cas9-induced indels in the *HNF1A* target sequence was affected by the patient-specific monoallelic frameshift mutation.

### gRNA testing in HepG2

To verify the gRNA editing efficiency in cells with intact *HNF1A*, HepG2 cells were used. HepG2 were cultured in RPMI 1640 medium (Gibco) supplemented with 10% fetal bovine serum (FBS; Gibco) and passaged after reaching 80% confluence. A total of 5 µg of PX459 plasmid encoding Cas9 and the candidate gRNA was electroporated into 2.0 × 10⁶ HepG2 cells using the Nucleofector IIb system (Lonza/Amaxa Biosystems) and the Human Stem Cell Nucleofector^®^ Kit 1 (Lonza), with the HepG2-dedicated T-028 electroporation program. A day later, cells were subjected to puromycin selection (1.5 µg/mL) for 24 h to enrich for transfected populations. Genomic DNA was isolated 96 h post-electroporation, and the targeted locus was PCR-amplified and analyzed by Sanger sequencing. Editing efficiency for each gRNA was analysed using the same tools employed during gRNA validation in hiPSCs.

### ssODN HDR template design

To maximize HDR efficiency, a 150-nucleotide ssODN repair template was designed (GGGGAGTCCTGCGGCGGCGGTCGAGGGGAGCTGGCTGAGCAGGACGAGACGGACGACGATTGCCCAATGGGCTGGGGGAGACTCGGGGCTCCGAGGACGAGACGGACGACGATGG**T** GAAGACTTCACGCCACCCATCCTCAAAGAGCTGGAGAACCTCAGCCCTGAGGAG). The ssODN featured asymmetric homology arms flanking the PAM site, with a 93-nt PAM-distal arm and a 54-nt PAM-proximal arm. To prevent the re-cleavage of a repaired allele by the CRISPR RNP complex, a silent G→T mutation was introduced at the PAM site (highlighted). The chemically modified ssODN (Alt-R™ HDR Donor Oligo) was synthesized by IDT.

### RNP electroporation and post-electroporation culture

As a first attempt 2.0 × 10⁶ hiPSCs were electroporated using a Nucleofector IIb (Lonza/Amaxa Biosystems) with a preassembled Cas9-GFP/sgRNA RNP complex and ssODN repair template, followed by clonal expansion, cryopreservation, and DNA-based screening of single-cell-derived colonies.

As the initial electroporation attempt did not yield any correctly repaired clones, 6.0 × 10⁵ hiPSCs were electroporated using the MaxCyte ExPERT GTx system with a preassembled Cas9-GFP/sgRNA RNP complex. The ssODN repair templates were tested at varying concentrations (2.5–10.0 µM), using the iPSC-S2 and iPSC-S4 programs. Post-electroporation, cells were cultured either with or without the DNA ligase IV inhibitor SCR7 to assess its effect on repair efficiency, while GFP-based positive controls were used to validate electroporation performance. Cells were subsequently clonally expanded, cryopreserved, and screened by DNA analysis following isolation of single-cell-derived colonies. More detailed information regarding the exact electroporation procedure is provided in the Supplementary Methods.

### Clone screening with surveyor assay

To evaluate mutation correction, initial screening was performed using the Surveyor nuclease, employing a homemade celery juice extract, as adapted from Henikoff et al. (Till et al. 2006). Before heteroduplex formation and enzymatic digestion, a portion of the PCR amplicons spanning the *HNF1A* mutation site was analysed by electrophoresis on a 2% agarose gel to confirm the presence of a single, specific product. The remaining amplicons were then denatured at 95 °C for 10 min, followed by gradual reannealing, with a 60-second incubation at each 10 °C decrement. The reannealed products were then incubated for 45 min at 45 °C with celery-derived homemade Surveyor nuclease. The digestion products were resolved by electrophoresis on a 2% agarose gel (70 V, 45 min) to visualize cleavage patterns indicative of genome editing. Clones displaying a single, intact band were considered candidates for successful correction and were subsequently subjected to Sanger sequencing to confirm precise genome modification and ensure sequence integrity.

### Immunocytochemical staining

To visualize targeted markers, cells were fixed with 4% paraformaldehyde (PFA; Sigma) at room temperature (RT) for 15 min, followed by three washes with PBS. For permeabilization, cells were incubated with 0.1% Triton X-100 in PBS for 15 min at RT, then blocked with 3% bovine serum albumin (BSA) in PBS for 1 h at RT. Cells were subsequently incubated overnight at 4 °C with the primary antibodies (Supplementary Table 4) diluted in 3% BSA in PBS. The following day, the cells were washed and stained with secondary (Supplementary Table 4) antibodies and together with DAPI diluted in 3% BSA in PBS for 1 h at RT. After incubation, cells were washed twice with PBS for 5 min each. High-resolution images were captured using Leica DMi8 fluorescence microscope.

### Assessment of *HNF1A* expression

qPCR was employed to evaluate *HNF1A* expression levels in the hiPSC lines and HepG2 as a positive control. First, total RNA was extracted from hiPSC cultures using the Total RNA Mini Kit (A&A Biotechnology) following the manufacturer’s protocol. RT-PCR was carried out according to the manufacturer’s instructions using 1 µg of total RNA as template with the High-Capacity cDNA Reverse Transcription Kit (Thermo Fisher Scientific) in a ProFlex PCR System (Thermo Fisher Scientific). qPCR amplification was conducted using SYBR Green Master Mix (Thermo Fisher Scientific) with 30 ng of cDNA template. Gene-specific primers were used for *HNF1A* and the housekeeping gene *EEF2* (Supplementary Table 1). The reaction was performed using a StepOne™ Real-Time PCR System (Applied Biosystems). Melt curve analysis was performed to verify the specificity of amplification. Relative gene expression was calculated using the 2^(−ΔΔCt)^ method, with *EEF2* serving as the internal reference gene for normalization.

### Assessment of the HNF1A protein expression

HNF1A expression was assessed in hiPSC-derived hepatoblasts, as baseline expression in undifferentiated hiPSCs is low. Briefly, hiPSCs were differentiated into hepatocyte-like cells using a previously published protocol (Mathapati et al. 2016), and the differentiation was terminated at the hepatoblast stage (after finishing stage II). Then, cells were lysed with RIPA buffer (Sigma) supplemented with protease inhibitor mix (Thermo Fisher Scientific). Protein concentration was determined using the bicinchoninic acid (BCA) assay. Twenty micrograms of protein were denatured and separated by SDS-PAGE, followed by wet transfer to methanol-activated PVDF membranes (Amersham). Membranes were blocked with 5% non-fat dry milk in 0.1% Tween-20 in Tris-buffered saline (TBS-T) for 2 h at room temperature, then incubated overnight at 4 °C with primary antibodies (anti-HNF1A antibody, or anti-GAPDH, Supplementary Table 4). After washing with TBS-T, membranes were incubated with HRP-conjugated secondary antibody for 1 h at room temperature (Supplementary Table 4). Protein bands were visualized using enhanced chemiluminescence substrate (Millipore) and detected with a ChemiDoc Imaging System (Bio-Rad).

### Statistical analysis

The HNF1A transcriptome and proteome analyses are presented as mean ± standard deviation (SD) from at least three independent biological experiments (*N* ≥ 3 independent hiPSCs differentiations or hiPSCs passages). Statistical significance was determined by one-way ANOVA using GraphPad Prism (GraphPad Software Inc., San Diego, CA, USA).

## Results

### Patient information

The hiPSCs were derived from peripheral blood mononuclear cells of a male patient with a c.235_236insG mutation in exon 1 of the *HNF1A*. The patient was diagnosed with HNF1A-MODY at the age of 18, whereas at the time of cell collection he was 27. The patient was treated with sulfonylurea, maintaining stable glycemic control. His BMI was normal (25.8), and at that time he showed no clinical signs of developing vascular diabetic complications like chronic kidney disease or retinopathy. The patient tested negative for HCV and HIV. The patient’s hiPSCs were used for the generation of the CRISPR/Cas9-mediated isogenic control line.

### gRNA design

To identify the most effective guide RNA (gRNA) for directing Cas9 to the precise *HNF1A* mutation site, an 80-nucleotide genomic sequence (40 nt upstream and 40 nt downstream of the mutation) was analysed. Since different publicly available tools employ distinct models to predict gRNA efficiency and off-target activity, three independent platforms—CRISPOR, CHOPCHOP, and Cas-Designer were used for cross-validation. The selection process prioritized specificity to minimize potential off-target effects, proximity to the mutation site to ensure efficient editing, and overall predicted general editing performance. Notably, the c.235_236insG mutation introduced a novel gRNA binding site (gRNA-2), which is absent in the wild-type *HNF1A* sequence, allowing allele-specific targeting of the mutant variant. Based on these criteria, three 20-nucleotide sgRNA candidates were selected for further evaluation.

Further analysis with CRISPOR revealed that all predicted off-target sites contained mismatches within the 12-bp seed region adjacent to the PAM, a key determinant of off-target activity. This pattern reduces the likelihood of unintended Cas9 binding and cleavage, further supporting the specificity of the selected sgRNAs.

### Engineering of plasmid gRNA-containing vectors, hiPSCs electroporation, and identification of the most promising gRNA

To evaluate the efficiency of the selected gRNAs, a plasmid-based approach was chosen as a cost-effective alternative to ribonucleoprotein (RNP)-mediated CRISPR delivery. The gRNA sequences were cloned into the *pSpCas9(BB)-2 A-Puro* (PX459) vector following the protocol described by (Santos et al. 2016) adapted from (Ran et al. 2013). After plasmid purification, the patient-specific hiPSCs were nucleoporated with the CRISPR-containing plasmid constructs using an Amaxa IIb nucleofector. To enrich the fraction of successfully nucleoporated cells, puromycin selection was applied on the next day and maintained for 24 h. Genomic DNA was then extracted, and the region containing the mutation site was amplified via PCR and analyzed by Sanger sequencing. The efficiency of each gRNA was evaluated using two sequence decomposition tools TIDE (Brinkman et al. 2018) and DECODR (Bloh et al. 2021). Among the three sgRNA candidates, gRNA-1 exhibited the highest apparent efficiency. However, the analysis was hindered by the nature of the monogenic frameshift mutation, which complicated the distinction between CRISPR-induced indels and mutation-associated frameshift.

To better evaluate sgRNA efficiency, we employed an alternative cellular model, as hiPSCs may be suboptimal due to their inherently low electroporation efficiency and high fragility (Li et al. 2018), which can obscure the true activity of gRNAs. Instead, we used the HepG2 cell line, which offered an effective system due to its high transfection efficiency and the absence of the disease-associated mutation. As a hepatic cell line, HepG2 also offers biological relevance for *HNF1A* since this gene is mainly expressed in hepatic cells, making it a practical platform for preliminary sgRNA validation. We tested gRNA-1 and gRNA-3 in HepG2 cells, but not gRNA-2, as its sequence specifically targets the patient-derived *HNF1A* mutant allele. The experimental conditions were consistent with those used for hiPSCs, including puromycin selection and reagents. The results from HepG2 sequencing showed that gRNA-1 had the highest editing efficiency (27.2% by TIDE; 21.9% by DECODR), with both tools identifying a statistically significant 3-nucleotide deletion and a 1-nucleotide insertion. In contrast, gRNA-3 showed lower efficiency (8.7% by TIDE; 4.7% by DECODR), with both tools detecting a significant 1-nucleotide deletion. Therefore, gRNA-1 was selected for gene correction using the RNP complex. While plasmid-based CRISPR delivery provided an initial screening method for gRNA selection, RNP-based gene editing was chosen for the final correction step due to its higher efficiency and lower cytotoxicity in hiPSCs (Liang et al. 2015; Han et al. 2024).

### ssODN HDR template design

In HDR-based precise genome editing, various types of repair templates can be used depending on the target site, the size of the desired modification, and the cellular repair environment. Among these, single-stranded oligodeoxynucleotides (ssODNs) are widely employed due to their high efficiency, lower risk of random integration, and greater suitability for hiPSC editing (Shakirova et al. 2023). Additionally, chemically modified templates can be used to improve stability and enhance HDR efficiency. 

**Fig. 1:**
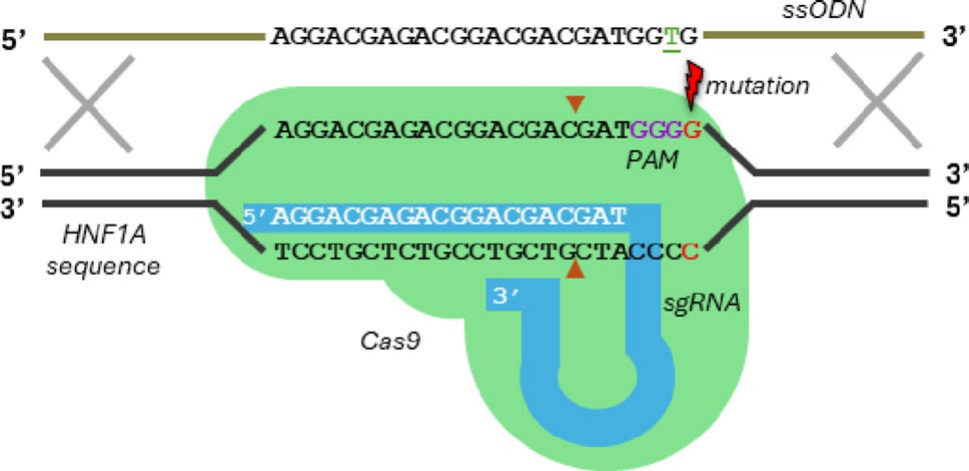
Schematic representation of CRISPR-based *HNF1A* correction in the HNF1A-mutated patient-specific hiPSC line using an RNP-ssODN complex. The used sequences of the selected gRNA-1 and ssODN are shown, with the silent mutation in the PAM region of the ssODN highlighted in green. The Cas9 cut site is indicated by orange triangles.

For this study, a 150-nucleotide ssODN repair template was designed with asymmetric homology arms flanking the PAM site, featuring a longer PAM-distal arm (relative to the protospacer adjacent motif, PAM) and a shorter PAM-proximal arm. This asymmetry plays an important role in enhancing HDR efficiency, as studies have demonstrated that asymmetric ssODNs consistently achieve higher repair rates compared to symmetric templates (Liang et al. 2017). This is thought to better mimic natural DNA repair intermediates, facilitating strand invasion and stabilizing the repair process (Lanza et al. 2018). To improve stability and resistance to nucleases, the ssODN was chemically modified using the Alt-R modification from Integrated DNA Technologies (IDT). To further prevent unintended cleavage of the repair template, a silent mutation was strategically introduced at the PAM site, ensuring that the Cas9:sgRNA complex could no longer recognize and cut either the ssODN template or the newly corrected genomic sequence (Ma et al. 2015). A schematic representation of the RNP with the PAM and ssODN is shown in Fig. 1.

### CRISPR-Cas9 repair of HNF1A-mutated hiPSCs

To enhance HDR efficiency, CRISPR components were introduced as an RNP complex, a delivery approach that increases editing efficiency and reduces prolonged Cas9 activity, thereby limiting off-target effects (Liang et al. 2015). Our initial strategy involved delivering the Cas9-GFP: sgRNA complex and ssODN into patient-derived hiPSCs using the Amaxa IIb nucleofector. Previous studies suggest that GFP-labeled Cas9 can facilitate the selection of successfully electroporated cells (Ye et al. 2021; Caillaud et al. 2022) and thus increase the chance of finding corrected clones. To check this, we performed single-cell sorting 24 h post-electroporation using the MoFlo XDP Cell Sorter (Beckman Coulter), aiming to enrich the fraction of successfully electroporated cells. Although we observed a population of GFP⁺ cells and optimized sorting conditions, such as minimizing sheath fluid speed and sorting into conditioned hiPSCs medium, colony survival post-sorting remained extremely low. Out of 288 single-sorted cells plated in four 96-well Geltrex-coated plates, none survived. As a backup, to enhance the survival by increasing cell density, we additionally sorted 150 GFP⁺ cells per well into ten wells of six-well Geltrex-coated plates. However, this approach resulted in only four surviving colonies, all of which harboured the patient-specific mutation, indicating that HDR-mediated repair had not occurred.

To increase the number of the tested clones, the electroporation was repeated without the sorting step, yielding approximately 50 clones for HDR screening. To efficiently manage the large number of clones requiring analysis, we implemented a pre-screening step using the Surveyor assay, enabling the cost-effective selection of the most promising candidates for Sanger sequencing. However, given the typically low efficiency of HDR, screening only 50 clones may have been insufficient to identify a successfully corrected one. Indeed, Sanger sequencing validation confirmed that none of the analyzed clones exhibited successful mutation repair.

### Optimization of the repair strategy

To improve cell survival after electroporation and increase colony yield, we switched to a MaxCyte’s ExPERT™ electroporator and tested two programs with varying electroporation energy. Additionally, we adjusted the ratio of components within the RNP complex and tested three ssODN concentrations. The small reaction volume of the MaxCyte system allowed for the simultaneous evaluation of multiple conditions.

In all conditions, hiPSCs were electroporated with an RNP complex consisting of sgRNA and Cas9-GFP at a 2:1 molar ratio, combined with the varying ssODN concentrations (2. 5, 5.0, and 10.0 µM). To further enhance HDR efficiency, half of the electroporated cells from each condition were cultured in a medium supplemented with SCR7 pyrazine, a DNA ligase IV inhibitor known to promote HDR by reducing the activity of the competing NHEJ pathway (Hu et al. 2018). In total, 12 different combinations were tested. A summary of the number of observed and screened clones in each of the twelve different conditions is provided in Figure S1A (Supplementary Figures).

Across all tested conditions, a total of 258 colonies were observed. The addition of SCR7 did increase the number of observed colonies, with 94 colonies observed without SCR7 and 164 with SCR7. Additionally, the higher-energy program iPSC-S4 resulted in a greater number of colonies (108 vs. 150), independent of the addition of SCR7. Among all detected colonies, 53 were electroporated with 2.5 µM ssODN, 98 with 5 µM ssODN, and 107 with 10 µM ssODN. However, out of 258 observed colonies only 113 clones in total could be successfully expanded and maintained in culture for at least three passages. These samples were initially screened using the Surveyor assay (Fig. S1B), enabling identification of the most promising clones with potential mutation correction. Out of the 113 pre-screened clones, 14 candidates were selected and sent for Sanger sequencing, corresponding to a pre-screening-to-sequencing rate of ~ 12.4%. The second stage of analysis resulted in the identification of five successfully repaired clones: one from condition 5, three from condition 6, and one from condition 11 (~ 4%). However, four of these five repaired clones carried additional unintended mutations in *HNF1A* or could not be kept in culture long term. Therefore, only one corrected clone from condition 5 could be used in further studies.

To assess whether electroporated cells could be cryopreserved for later screening, the remaining cells from condition 11 (Fig. S1A) were thawed and expanded on a 6-well plate. This condition was selected because it initially exhibited the highest number of observed colonies, which could indirectly suggest greater stability and higher viability of electroporated cells under these specific parameters, particularly important for withstanding the additional stress imposed by cryopreservation and thawing. Subsequently, 1000 cells per dish were seeded on three 10 cm culture dishes. This approach led to the isolation of 60 additional clones, all of which were successfully propagated and pre-screened. Eighteen (18) of these 60 pre-screened clones were sent for sequencing, and after analysis three corrected clones were identified. However, only one clone retained precise correction without additional mutations and thus could be used for further experiments. These results confirm that hiPSCs can be cryopreserved for approximately six months post-electroporation and successfully used for later colony screening, increasing the number of the tested clones.

**Fig. 2:**
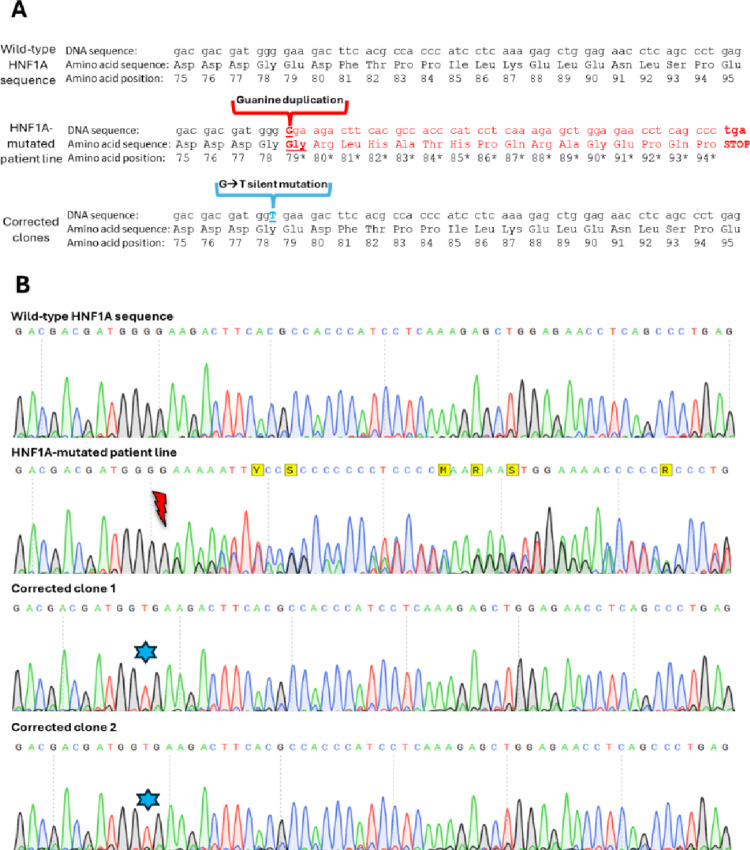
**A** Schematic representation of *HNF1A* sequences, including the wild-type, the heterozygous HNF1A-mutated patient-specific line, and the repaired clones. In the patient-specific line, the mutated DNA sequence and corresponding amino acid changes are highlighted in red, with affected amino acid positions marked by asterisks (*). **B** Sanger sequencing results corresponding to the *HNF1A* region shown in panel A. The patient-specific mutation is marked in red, while the silent mutation introduced in the repaired clones is indicated by a blue asterisk.

Summarizing our approach of correction of the patient-specific *HNF1A* mutation using the MaxCyte electroporation system, a total of 173 clones (113 from all conditions + 60 from cryopreserved cells from condition 11) were established and pre-screened with the Surveyor assay, identifying 32 candidates for Sanger sequencing (~ 18.5% overall pre-screening-to-sequencing rate). Among these, 24 clones retained the original c.235_236insG mutation and/or acquired additional mutations due to NHEJ. Eight clones exhibited successful HDR-mediated correction, where the pathogenic c.235_236insG mutation was replaced with the intended sequence. The corrected clones incorporated a heterozygous silent substitution (c.G234T; GGG → GGT) due to recombination with the ssODN donor template (Fig. 2A, B). However, only two clones showed precise correction without additional indels that could affect HNF1A protein function, restoring the reading frame. Therefore, for subsequent experiments, the repaired clones obtained under conditions 5 and 11 (for condition 11 the repaired clone was identified after analysis of cells thawed from frozen stock) were designated as Repaired Clone 1 and Repaired Clone 2, respectively.

### Off-target analysis

To assess potential off-target effects, the two corrected hiPSC clones were screened for unintended gRNA activity. Off-target sites were predicted in silico using CHOPCHOP and CRISPOR. CRISPOR showed a total of 22 potential off-target sites. Among these, a site in the intronic region of the *GSE1* gene (chr16:85614991) contained three mismatches, while the remaining 21 sites had four mismatches. As CHOPCHOP detects only off-targets with three or fewer mismatches, it identified only the *GSE1* site. Given the lowest mismatch count which indicates the highest chance of unintended off-target activity, this site was prioritized for experimental validation as the most likely off-target candidate.

To confirm the specificity of the CRISPR/Cas9 editing approach, Sanger sequencing was performed on the *GSE1* locus in both corrected hiPSC clones. The analysis revealed no unintended genome modifications, further supporting the precision and reliability of the genome editing strategy (Fig. S2).

### Pluripotency characterization of the repaired hiPSC clones

Ensuring that genetically corrected hiPSCs maintain pluripotency is essential in CRISPR-based gene correction studies, as unintended genetic modifications could compromise their differentiation potential and limit their utility in disease modelling applications. To verify that CRISPR-edited hiPSCs retained their pluripotency and differentiation capacity, immunofluorescence staining was performed for key pluripotency markers, accompanied by a trilineage spontaneous differentiation assay.

**Fig. 3:**
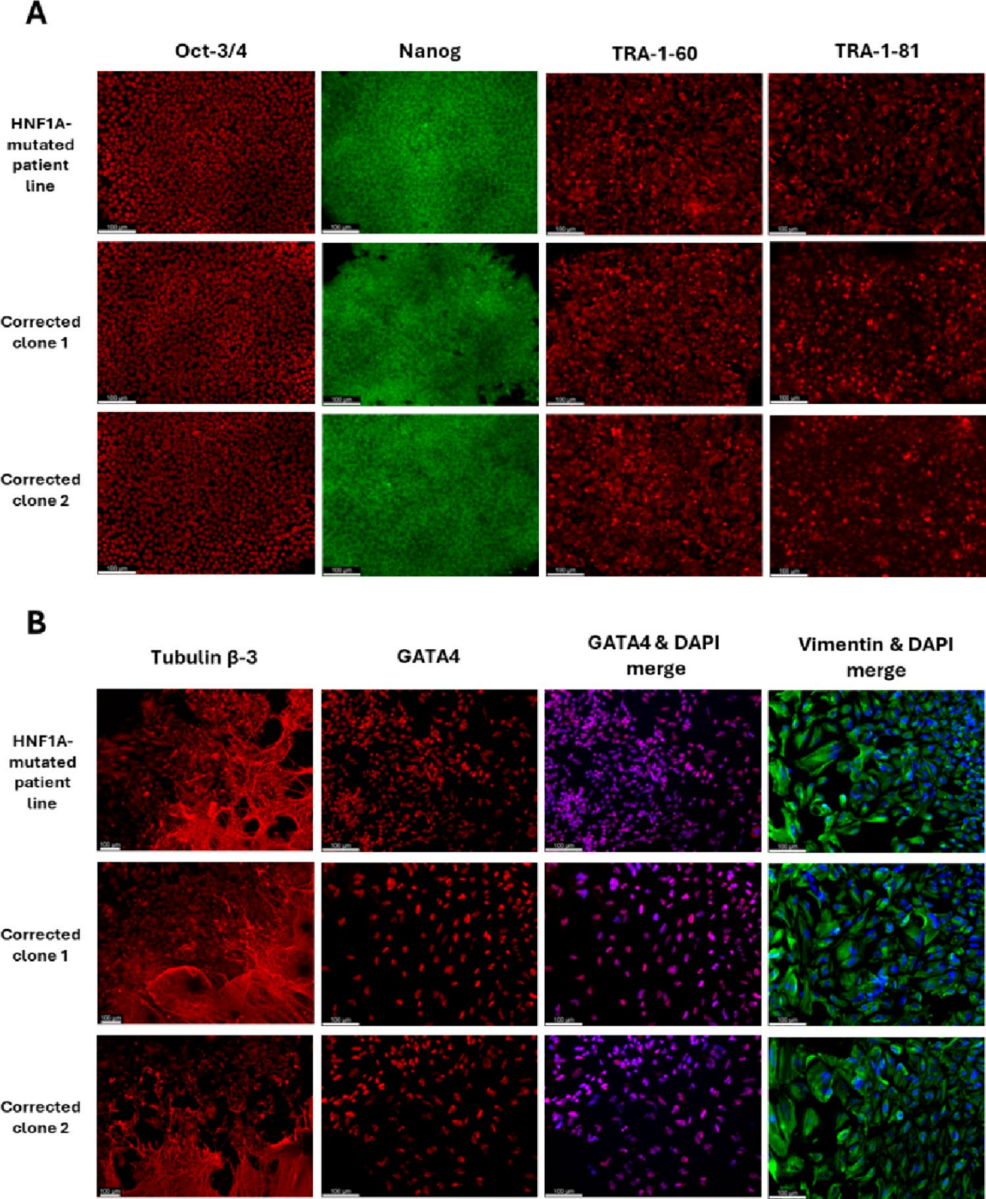
**A** Immunofluorescent staining of pluripotency markers in hiPSC lines, including the HNF1A-mutated patient-specific line and both corrected clones. OCT-3/4 (red), NANOG (green), TRA-1-60 (red), and TRA-1-81 (red) are shown in their respective panels. **B** Spontaneous *in vitro* differentiation of hiPSC lines via embryoid body formation, analyzed by immunocytochemistry for lineage-specific markers representing the three embryonic germ layers. Endoderm is visualized with GATA4 (red), mesoderm with Vimentin (green), and ectoderm with Tubulin β-3 (red). Nuclei are counterstained with DAPI (blue).

Immunofluorescence analysis of undifferentiated hiPSCs confirmed the expression of core pluripotency markers, including NANOG, OCT-3/4, TRA-1-60, and TRA-1-81, indicating that genome editing did not affect their fundamental stem cell identity (Fig. 3A). To assess their differentiation potential, an embryoid body (EB) spontaneous differentiation was conducted. Immunofluorescence staining demonstrated robust expression of lineage-specific markers, including GATA4 (endoderm), Vimentin (mesoderm), and Tubulin-β3 (ectoderm), confirming that the corrected hiPSC clones retained the ability to generate derivatives of all three germ layers (Fig. 3B). Subsequent tests of the hiPSCs confirmed genomic identity of the repaired clones with the parental line, their normal karyotype, absence of reprogramming vector, lack of mycoplasma contamination, and HIV genome integration (Supplementary material 2).

### Effect of the mutation repair on HNF1A expression levels

**Fig. 4:**
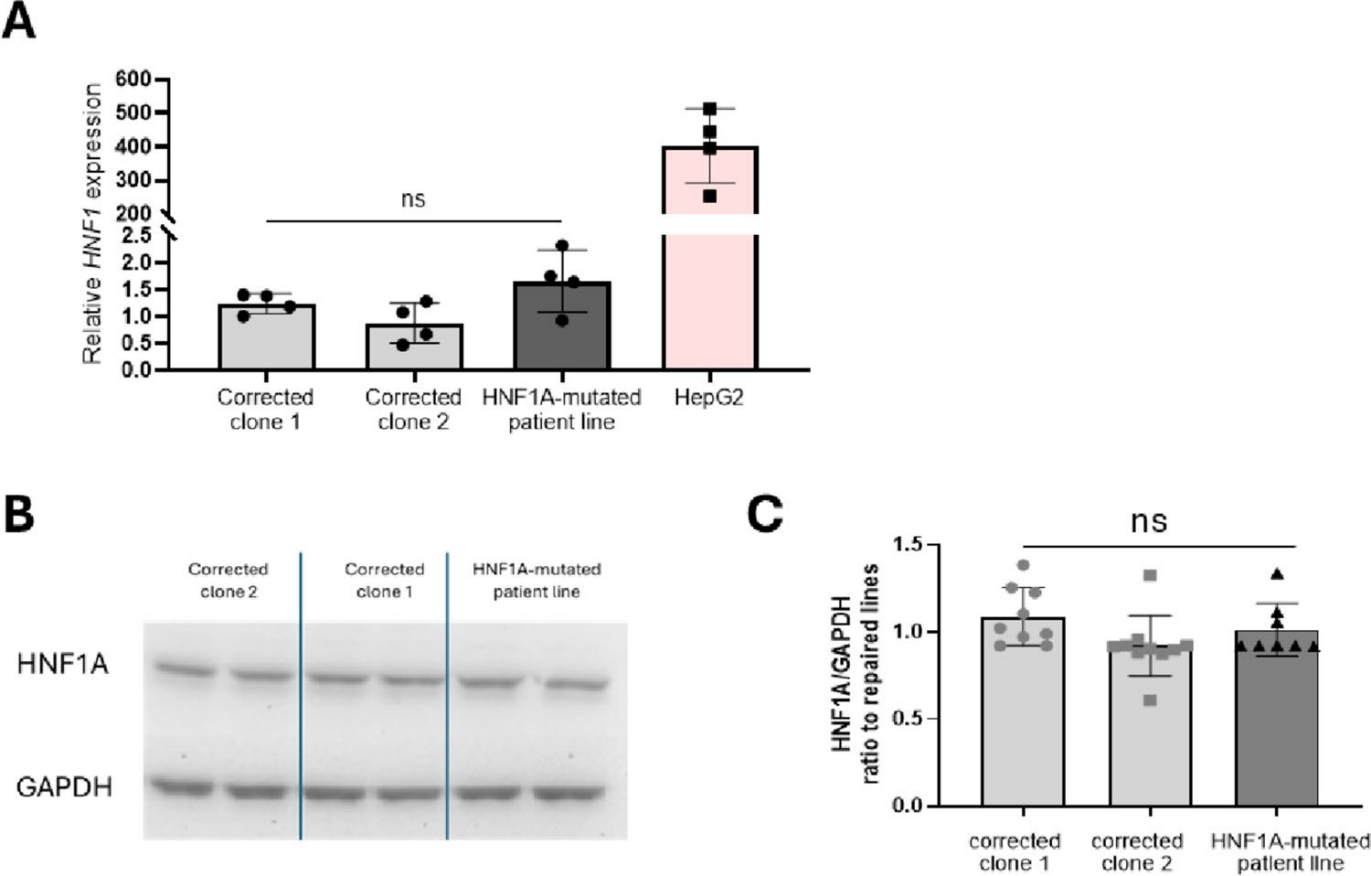
**A** Relative *HNF1A* mRNA expression levels determined by qPCR in hiPSC clones. *EEF2* was used as the housekeeping gene for normalization, and HepG2 cells served as a positive control for *HNF1A* expression. **B** Representative Western blot analysis of HNF1A; **C** Western blot analysis of HNF1A expression. Densitometric signals were normalized to GAPDH. Data are presented as fold change relative to the mean of both corrected control clones. Statistical significance was determined by one-way ANOVA, confirming lack of difference in the HNF1A expression between the tested lines.

Analysis of *HNF1A* expression on both mRNA (Fig. 4A) and protein levels (Fig. 4B, C) did not show changes in the corrected clones, as compared to the *HNF1A*-mutated patient line. This is in line with our recent results, where we compared the protein levels of HNF1A from the *HNF1A*-mutated patient line to healthy donor hiPSCs lines (Skoczek et al. 2026). Even though no significant changes on protein or transcriptome level could be detected, the repair of *HNF1A* mutation in the patient-derived hiPSCs had functional consequences in the hiPSC-derived endothelial cells. We could show that the endothelial cells with repaired *HNF1A* mutation restored their glycocalyx layer in comparison to the mutated patient line, which was in line with the results obtained with a different isogenic set of lines (Skoczek et al. 2026). Moreover, we have performed global transcriptome analysis of these patient-specific hiPSC-derived endothelial cells (ArrayExpress E-MTAB-16548). The Gene Set Enrichment Analysis (GSEA) using the Reactome database identified the top 100 enriched pathways based on Wald statistics. The major functional modules included cell cycle, extracellular matrix (ECM) organization, glucose metabolism, interleukin signalling, TLR signalling, and death receptor signalling (Skoczek et al. 2026). Ridgeplot analysis of representative pathways revealed predominant downregulation of ECM-related pathways and upregulation of immune-related pathways, cell cycle, and glucose metabolism. These findings were consistent with those observed in the control-derived isogenic set (Skoczek et al. 2026).

## Discussion

hiPSCs have become a fundamental tool in disease modeling, particularly when combined with CRISPR/Cas9 gene-editing technologies. However, for rare monogenic disorders such as HNF1A-MODY, patient-specific gene correction in hiPSCs remains limited, with only a few documented cases (Cujba et al. 2022; González et al. 2022; Hermann et al. 2023). Given the genetic heterogeneity of HNF1A-MODY, which could arise from diverse pathogenic variants dispersed throughout the *HNF1A* gene, establishing multiple isogenic hiPSC lines is crucial for accurately modeling the disease. These lines can facilitate the correlation of specific mutations with disease progression, providing a valuable platform for mechanistic studies and therapeutic testing. Although Sanger sequencing confirmed successful genomic correction, *HNF1A* expression levels remained unchanged in the corrected clones. In the mutated line, the remaining wild-type *HNF1A* allele appears sufficient to maintain normal expression levels, effectively masking the haploinsufficiency. Although premature stop codons frequently trigger nonsense-mediated mRNA decay (NMD), some exceptions have been described. One of the most extensively studied frameshift mutations in *HNF1A* is p.Pro291GlnfsTer51 (ClinVar Variation ID 435424). However, its precise mechanism of action remains incompletely understood. In an early study by Yamagata et al. (1998), it was suggested that the p.Pro291GlnfsTer51 mutation functions as a dominant-negative variant in HNF1A. Subsequently, others concluded that p.Pro291GlnfsTer51 primarily acts through haploinsufficiency rather than a dominant-negative mechanism (Harries et al. 2004). More recently, a human induced pluripotent stem cell (hiPSC) line with a heterozygous p.Pro291GlnfsTer51 mutation was generated using CRISPR/Cas9 (Cujba et al. 2022). In this model, the *HNF1A* mRNA expression remained unchanged in both pancreatic progenitor cells and in pancreatic organoids as compared with wild-type controls. The authors proposed that the truncated HNF1A protein produced by the p.Pro291GlnfsTer51 mutation may disrupt heterodimer formation between HNF1A and HNF1B, thereby altering the transcription of downstream genes. Collectively, these findings suggest that the pathogenic mechanism of p.Pro291GlnfsTer51 is multifaceted, likely involving both transcript instability and impaired protein–protein interactions. A comparable mechanism may underlie the frameshift mutation identified in the present study. Importantly, our recent findings demonstrate that correction of the *HNF1A* mutation restores the glycocalyx layer in hiPSC-derived endothelial cells. In this study, we compared this patient-derived isogenic hiPSC set with a second isogenic model in which a mutation in *HNF1A* was introduced. Consistently, both models exhibited concordant alterations in the glycocalyx, with *HNF1A*-mutant endothelial cells showing a reduced glycocalyx layer. A comparable effect was observed in primary endothelial cells following siRNA-mediated reduction of *HNF1A* expression. Furthermore, global transcriptomic analyses across both isogenic systems revealed comparable gene expression changes (Skoczek et al. 2026), affecting pathways related to cell cycle regulation, ECM organization, glucose metabolism, and interleukin signalling, further supporting the functional impact of *HNF1A* correction in patient-derived hiPSCs.

**Fig. 5:**
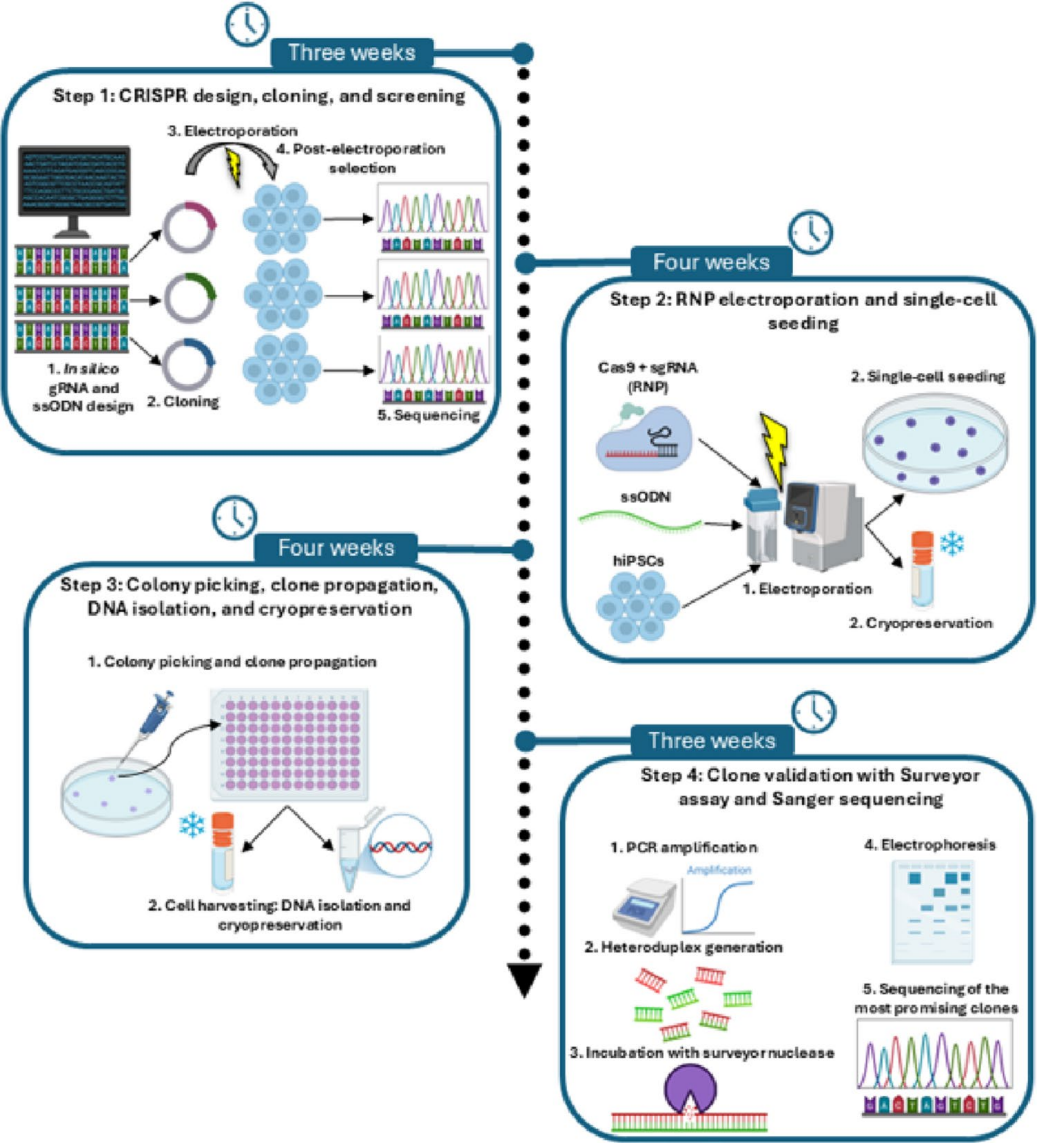
Schematic representation of the CRISPR-based mutation-correction pipeline, highlighting the four key steps in the process. Finally, off-target effects of CRISPR/Cas9 editing were evaluated only using in silico prediction tools, which provide limited resolution and may overlook unexpected genomic alterations. More comprehensive and unbiased approaches, such as GUIDE-seq, DISCOVER-seq, or ChIP-seq-based methods, would offer a more robust assessment of genome-wide off-target activity in the corrected clones, (Kalter et al. 2025).

While our study successfully generated an HNF1A-MODY patient-specific hiPSC line via CRISPR/Cas9-mediated correction of the heterozygous c.235_236insG mutation, several challenges emerged that highlight critical considerations for precision gene editing in hiPSCs (Fig. 5). A major limitation remains the low editing efficiency of these cells, with only ~ 4.5% of all screened clones exhibiting mutation repair. However, with only two clones that were precisely corrected and suitable for further studies, the overall efficiency drops to ~ 1%. This highlights the necessity of screening large numbers of clones to identify accurately edited ones, making the gene editing process labor-intensive and time-consuming. It is known that the HDR efficiency in hiPSCs is often low due to a combination of cell-cycle constraints, DNA repair pathway bias, and cellular stress responses. HDR is restricted primarily to the S and G2 phases, while hiPSCs frequently favor the faster NHEJ, which competes with and suppresses precise repair (Shams et al. 2022). Additionally, delivery inefficiencies of donor templates and genome-editing components can limit HDR engagement. hiPSCs are also highly sensitive to DNA damage, activating p53-mediated responses that can induce cell cycle arrest or apoptosis, thereby reducing the pool of successfully edited cells (Park et al. 2024a). Epigenetic context and chromatin accessibility at the target locus further influence repair outcomes, often making certain genomic regions less amenable to HDR (Yarrington et al. 2018). Thus, the sgRNA efficiency was assessed in a more permissive cell model, a HepG2 cell line, which is unrelated to the patient but offers significantly higher transfection efficiency. This approach allowed us to pre-screen and identify the most effective sgRNA under optimized conditions before applying it to patient-derived hiPSCs. Such intermediate validation in a robust, easily transfected system can serve as a valuable strategy for streamlining the optimization process while minimizing the influence of confounding variables prior to implementation in more sensitive cell types like hiPSCs. Electroporation remains the most effective method for delivering CRISPR into hiPSCs. However, the process is inherently stressful to these cells, which are particularly sensitive to physical manipulation. Therefore, efforts should also focus on fine-tuning electroporation parameters to enhance delivery efficiency while preserving cell viability, ultimately improving overall editing outcomes in this challenging yet powerful model system.

These and other factors highlight the need for improved strategies to enhance editing efficiency, maintain clonal stability, and streamline screening. Addressing these challenges is crucial for ensuring the reliability and reproducibility of CRISPR-based gene correction in hiPSCs. Based on our experience, in the next section, we will discuss the most critical challenges in CRISPR-based mutation repair, outlining key aspects that should be carefully considered when designing and optimizing such experiments.

### Challenges and considerations in CRISPR/Cas9 repair strategies for mutation correction in hiPSCs

#### Delivery of the CRISPR components into hiPSCs

CRISPR delivery methods significantly impact genome-editing outcomes, with electroporation, viral vectors, and lipofection being the most commonly used approaches (Du et al. 2023). Among these, electroporation is highly effective, particularly for RNP-based CRISPR delivery, as it enables direct transport of CRISPR components into the nucleus, enhancing editing efficiency while minimizing prolonged Cas9 expression and subsequent off-target effects (Atri et al. 2021). In contrast, plasmid-based delivery leads to longer Cas9 expression, increasing the risk of off-target modifications, while viral vectors offer stable integration but pose challenges in precise genome editing due to persistent expression and potential immunogenicity (Du et al. 2023).

Various electroporation platforms, including the CTS Xenon Electroporation System (Thermo Fisher), MaxCyte^®^ ExPERT™ electroporation platforms, and Lonza Nucleofector^®^ IIb and 4D-Nucleofector^®^ System, offer efficient CRISPR delivery solutions. However, these systems differ in electroporation parameters such as voltage, pulse duration, and cell handling conditions, which may require prior optimization. Additionally, the scale varies across platforms, influencing the total amount of CRISPR reagents delivered per reaction, which can impact editing efficiency and reagent costs. Cell survival after electroporation remains a major concern, particularly for hiPSCs, which are highly sensitive to physical stress. Variability in survival rates across different hiPSC lines further emphasizes the need for optimized electroporation parameters, cell density, and post-electroporation culture conditions to balance both editing efficiency and cell viability.

In the current study, electroporation of hiPSCs using the Lonza Nucleofector II was unsuccessful, primarily due to reduced cell survival, particularly after cell sorting. However, even without the sorting step, a low number of colonies could be propagated, and none of them was repaired for the *HNF1A* mutation. Interestingly, a side-by-side comparison of the 4D Lonza nucleofection platform with MaxCyte in a hiPSCs knock-in experiment showed increased editing efficiency and cell viability with the MaxCyte system (Kagita et al. 2021). Given the variability in performance across different systems, pre-experimental literature screening and pilot testing of electroporation conditions are highly advisable. Evaluating multiple electroporation programs and, if feasible, different electroporation platforms using a fluorescent marker such as GFP can help assess transfection efficiency and cell viability before proceeding with the main CRISPR experiment. This preliminary optimization step is crucial for identifying conditions that maximize genome-editing efficiency while minimizing cytotoxicity.

#### Selection strategy: considerations for Cas9-GFP sorting

To facilitate the selection of successfully transfected cells, we employed Cas9-GFP fusion proteins, a strategy frequently recommended in the literature (Caillaud et al. 2022; Shahryari et al. 2021; Namipashaki et al. 2023). However, this approach proved completely ineffective in our study, as sorted cells exhibited poor survival, both when plated as single cells in 96-well plates and as a bulk population in 6-well plates. The mechanical stress induced by FACS, together with post-electroporation stress, significantly reduced viability in this hiPSCs line. Indeed, according to the literature, single-cell sorting of hiPSCs can reduce viability by up to 50% (Caillaud et al. 2022).

Cas9-GFP fusion proteins can also contribute to compromising editing efficiency. It has been reported that the Cas9-GFP fusion protein may reduce gene modification efficiency up to twofold compared to non-fused Cas9 (Nasri et al. 2019). Given the inherently low efficiency of HDR, any further reduction presents a significant limitation. While sorting the brightest GFP+ population may seem like a tempting approach to select cells with the highest CRISPR component uptake, it may be misleading, as these cells are also the most likely to experience increased cytotoxicity due to Cas9-GFP accumulation (Huang et al. 2021). Instead, selecting an intermediate GFP+ population might be more beneficial, balancing sufficient CRISPR component presence while reducing toxicity-associated stress. Based on these findings, we do not recommend Cas9-GFP-based selection for hiPSCs gene editing unless the specific cell line demonstrates high post-FACS survival.

Given the limitations associated with Cas9-GFP-based FACS in hiPSCs, it is worth considering alternative enrichment strategies. However, before implementing any selection-based approach, it is advisable to first optimize the core parameters of the genome editing protocol to achieve the highest possible HDR efficiency. In many cases, improvements to the delivery conditions or CRISPR components, such as sgRNA design, chemical modifications, or electroporation parameters, may be sufficient to yield edited clones without additional enrichment. If these baseline optimizations do not result in satisfactory HDR levels, applying an enrichment strategy may significantly increase the likelihood of recovering corrected clones. Several options are available, ranging from transient antibiotic selection using non-integrating or excisable resistance cassettes, which offer a reversible and scalable method of enrichment, to more advanced approaches. These include co-targeting endogenous selectable loci such as hypoxanthine phosphoribosyltransferase (HPRT) or α_1_N, K-ATPase (ATP1A1), enabling small-molecule-based selection, as well as episomal surrogate reporters that transiently mark nuclease activity without introducing permanent genomic modifications. Such strategies are particularly valuable in hiPSCs, where reducing cellular stress and preserving clonal integrity are essential for downstream applications (Mikkelsen and Bak 2023).

#### Increasing HDR efficiency

In our attempt at *HNF1A* correction, we applied SCR7 pyrazine, a DNA ligase IV inhibitor known to promote HDR, but observed no significant improvement in repair efficiency. While some studies report HDR frequency increases of 1.4- to 1.6-fold (Maurissen and Woltjen 2020), its overall efficacy remains controversial. Given the low basal HDR efficiency we encountered, such a modest enhancement may be insufficient to yield a meaningful impact. Interestingly, a higher number of colonies was observed after SCR7 addition, suggesting higher cell viability, although this may have occurred incidentally rather than as a direct effect of the compound.

Furthermore, studies suggest that SCR7 may not be a selective or potent DNA ligase IV inhibitor, which raises questions about its real efficacy and suitability for this application (Greco et al. 2016). The other factors that could affect our results are timing and dosage, and alternative regimens, such as pretreatment, which might yield better results. As HDR primarily occurs during the S/G2 phases and hiPSCs have a distinct cell cycle profile, SCR7 alone may not be adequate (Mao et al. 2008).

Emerging evidence suggests that SCR7 is more effective when combined with HDR-enhancing compounds, such as RAD51 stimulators (RS-1) (Park et al. 2024b) or DNA-PK (DNA-dependent protein kinase catalytic subunit) inhibitors (e.g., NU7441) (Robert et al. 2015). These combinatorial approaches have demonstrated greater success in improving precise genome editing outcomes compared to SCR7 alone. Recently, the design parameters of the ssODN donor template, including homology arm symmetry, CRISPR/Cas-blocking mutations, and strand complementarity were shown to significantly influence the HDR outcomes (Baum et al. 2026). Therefore, based on the current literature and our experience, a multifaceted strategy for improving HDR efficiency in hiPSCs is recommended including a combination of HDR promoting compounds and appropriate ssODN template design.

#### Clonal selection and expansion strategies

Although the importance of proper clonal selection is often overlooked in the literature, it plays a crucial role in generating stable and genetically modified hiPSC lines. Even in our best electroporation attempt, 145 out of the 258 observed colonies were lost (56%). However, for cells that underwent freezing and thawing, implementing handling optimizations, such as improving the timing of colony picking, allowed for the successful analysis of all recovered colonies. This highlights the necessity of an efficient and systematic clonal selection process to maximize the recovery of successfully edited clones and ensure reliable downstream analysis.

Our findings indicate that the timely selection of single-cell-derived hiPSC colonies is essential, as prolonged culture increases the risk of spontaneous differentiation. Colonies should be picked as soon as they become visibly distinct, even if this results in the loss of some colonies. Based on our experience, the optimal window for transferring single-cell expanded colonies is within 10–14 days post-seeding.

To support optimal expansion while maintaining pluripotency, a stepwise transition from smaller to larger well formats, progressing from 96-well to 48-well, then 24-well, and finally 12-well plates, is recommended. Additionally, cryopreservation at two key stages can enhance the availability of successfully corrected clones, particularly if the initial attempt fails (Fig. 5). The first cryopreservation time point can be introduced after single-cell seeding, utilizing leftover cells for future re-expansion and increasing the chances of obtaining correctly edited clones. The second time point may take place once the corrected clone has sufficiently expanded, ensuring a stable and reliable supply of modified cells for downstream applications. These cryopreservation steps not only increase the likelihood of recovering genetically corrected clones but also preserve their stability for further analysis, making them an integral part of a standardized clonal selection strategy.

#### Pre-screening of clones: considerations for surveyor assay

The Surveyor assay is a versatile and cost-effective tool that remains remarkably useful for screening multiple clones, despite having been somewhat overlooked in recent years. Its strengths lie in its broad applicability. It can be effectively employed to detect both induced mutations and corrections, making it an attractive option for high-throughput pre-screening that helps to significantly reduce the need for exhaustive Sanger sequencing. Nonetheless, the assay is not without its challenges that first need to be optimized. For instance, the cleavage products generated during electrophoresis can often appear faint, complicating their interpretation. In some instances, clones that initially appear to be corrected according to the Surveyor assay have later been confirmed to still harbor mutations upon Sanger sequencing, highlighting the risk of false negatives. Additionally, in cases involving the introduction or deletion of short sequences, the electrophoretic products of successfully edited clones may not differ significantly from the wild-type, potentially obscuring the experimental outcome. Moreover, the assay may not be applicable when PCR conditions are not optimal. This is particularly relevant when using high-fidelity polymerases, whose 3′→5′ exonuclease activity can inadvertently “correct” genuine mutations and lead to false-negative outcomes (Vouillot et al. 2015). Additionally, variations in proprietary PCR buffers can interfere with the activity of the Surveyor nuclease, further impacting assay accuracy.

To ensure the reliability of results, we recommend performing electrophoresis on a portion of the PCR product prior to heteroduplex formation and enzymatic digestion. This preliminary step verifies the presence of a single, specific amplicon and helps eliminate potential interference from nonspecific PCR products, thereby reducing the risk of misleading cleavage patterns. Additionally, careful selection of PCR reagents is crucial for assay accuracy. Based on our experience, the KAPA2G Fast HotStart PCR Kit (Roche) offers low error rates and optimal compatibility with the Surveyor assay. This reagent choice not only minimizes the risk of unintended mutation correction or introduction but also eliminates the need for additional gel loading buffers, streamlining the high-throughput screening process.

### Alternative genome editing strategies

Beyond conventional CRISPR/Cas9-mediated double-strand break repair, newer gene editing platforms such as base editors and prime editors offer promising alternatives for hiPSCs (Gao et al. 2025). Base editors, which couple a catalytically impaired Cas protein with a deaminase enzyme, enable precise nucleotide conversions (e.g., C→T or A→G) without inducing double-strand breaks, thereby reducing the risk of indels and improving editing fidelity in sensitive cell types like hiPSCs. Prime editors further expand this capability by combining a Cas9 nickase with a reverse transcriptase and a prime editing guide RNA (pegRNA), allowing for targeted insertions, deletions, and all possible base substitutions without relying on donor templates or double-strand breaks (Cerna-Chavez et al. 2025). These approaches are particularly advantageous in hiPSCs, where maintaining genomic integrity is critical, although their efficiency can still be influenced by chromatin accessibility, target site context, and delivery constraints.

### Limitations of the study

Several limitations of the current study should be acknowledged. First, all experiments were conducted using a single patient-derived hiPSC line, which constrains the generalizability of the findings and limits broader conclusions regarding the efficiency and reproducibility of the proposed repair strategy across diverse genetic backgrounds hiPSC lines.

Second, locus-specific factors may have influenced the efficiency of genome editing. In particular, the chromosomal context of HNF1A including local chromatin architecture, nucleosome occupancy, and epigenetic state. This can significantly affect the accessibility of the target DNA to the CRISPR/Cas9 machinery and the HDR pathway (Janssen et al. 2019). In hiPSCs, DNA accessibility is highly dynamic and regulated by chromatin remodeling, histone modifications, and DNA methylation. Regions of closed chromatin (heterochromatin) or strong nucleosome positioning can hinder Cas9 binding and cleavage, as well as the recruitment of HDR factors, thereby reducing repair efficiency (Yarrington et al. 2018). Conversely, open chromatin regions are generally more permissive to both Cas9 activity and template-directed repair. Thus, variability in chromatin accessibility at the HNF1A locus may have contributed to differences in editing outcomes.

## Conclusions

This study demonstrates the successful CRISPR/Cas9-mediated correction of an HNF1A-MODY patient-derived hiPSC line, providing a valuable model for studying monogenic diabetes. Beyond the technical achievement, our study highlights key challenges in Cas9-GFP selection, clonal expansion strategies, and Surveyor assay optimization, all of which are critical for enhancing editing efficiency and ensuring the successful application of hiPSC-based disease modeling.

Given the inherent limitations of conventional CRISPR/Cas9 editing, particularly the reliance on error-prone NHEJ and low-efficiency HDR, future studies may benefit from next-generation genome-editing tools, such as base and prime editors, which enable precise nucleotide modifications without introducing double-strand breaks. These advancements could further improve the accuracy, efficiency, and applicability of CRISPR-based gene correction in hiPSCs, facilitating more robust models for studying monogenic diseases and exploring therapeutic interventions.

## Supplementary Information

Below is the link to the electronic supplementary material.

Supplementary Material 1

Supplementary Material 2

## Data Availability

No datasets were generated or analysed during the current study.

## Declarations

### Figure preparation

Figures 1 and 2 A were created using Microsoft Office PowerPoint. Figure S1A was generated using the R programming environment. Figures S1B, S5 (gel electrophoresis), and 4B (PVDF membrane) are based on images captured using the ChemiDoc system (Bio-Rad). Figures 2B and S2 include Sanger sequencing chromatograms visualized with SnapGene Viewer (Insightful Science; available at https://www.snapgene.com). Figure 3 includes fluorescence microscopy images acquired using LAS X software (Leica Microsystems). Figure 4A and C were prepared with GraphPad software. Figure 5 was created with BioRender (https://biorender.com/kp31nos). All figures (1–5) were assembled using Microsoft Office PowerPoint.
