# Supplementary material for: CRISPR/Cas9-based repair of a heterozygous HNF1A mutation in patient-derived hiPSCs
Source: Hum Genet. 2026 Aug 12;145(1):69. doi: 10.1007/s00439-026-02863-0 (PMC13469492; doi:10.1007/s00439-026-02863-0)
Supplement: Supplementary file 1 [file 439_2026_2863_MOESM1_ESM.pdf]

CRISPR/Cas9-based repair of a heterozygous HNF1A mutation in patient-derived hiPSCs

Dawid Skoczek[1,2], Jerzy Hohendorff[3], Maciej T. Malecki[3], Alicia Roig-Merino[4], Rasmus O. Bak[5], Neli Kachamakova-Trojanowska[1]*

Supplementary Materials and Methods

Details on RNP electroporation and post-electroporation culture

Attempt one – Lonza IIb electroporation approach:

Electroporation was performed using an Nucleofector IIb (Lonza/Amaxa Biosystems), where the RNP complex (sgRNA: 5 µM, Cas9-GFP: 1 µM, 5:1 molar ratio) was preassembled for 20 minutes before being combined with 5 µM ssODN repair template, 2.0 × 10^6 hiPSCs and the components of the Human Stem Cell Nucleofector® Kit 1 (18 µL Supplement 1 and 82 µL Nucleofector Solution; Lonza). Electroporation was carried out using the B0-16 program, and cells were plated into a Geltrex-coated 6-well plate. Three days post-electroporation, cells were harvested, and 1,000 cells were seeded onto a Geltrex-coated 10 cm culture dish. After approximately two weeks of culture, single-cell-derived colonies were manually picked, transferred individually to Geltrex-coated 96-well plates, and expanded. Once sufficient cell numbers were obtained, clones were cryopreserved, and DNA was isolated from a subset for further screening. The sgRNA and Cas9-GFP were provided by IDT.

Attempt two – MaxCyte electroporation approach:

Electroporation was also performed using a MaxCyte^{TM} ExPERT GTx® system. The RNP complex was prepared by 20 minutes incubation of the components (sgRNA: 3 µM, Cas9-GFP: 1.5 µM, 2:1 molar ratio) at room temperature. Then the RNP was combined with ssODN repair templates at varying concentrations (2.5 µM, 5.0 µM, and 10.0 µM) and 6.0 × 10^5 hiPSCs, resuspended in MaxCyte electroporation buffer, to a final volume of 20 µL. Electroporation was performed using iPSC-S2 and iPSC-S4 programs. Positive controls, including mRNA GFP and plasmid-encoded GFP, were used to validate electroporation performance. The sgRNA and Cas9-GFP were provided by IDT.

After electroporation, cells were rinsed with 30 µL of MaxCyte buffer and plated into Geltrex-coated 96-well plates. Following a 10-minute recovery in a humidified incubator (37 °C, 5% CO_2), 50 µL of E8 medium supplemented with ROCKi. To evaluate repair efficiency with SCR7 pyrazine (DNA ligase IV inhibitor), half of the electroporated cells were transferred to Geltrex-coated 6-well plates containing 2 mL of E8 medium supplemented with ROCKi, while the remaining cells were cultured in E8 medium supplemented with ROCKi and 1.0 µM SCR7 (MedChemExpress). All cultures were maintained in a humidified incubator (37 °C, 5 % CO_2). After 48 hours, the medium was replaced with E8 medium without inhibitors. On day four, cells were harvested, and 1,000 cells from each condition were seeded onto Geltrex-coated 10 cm culture dishes, while the remaining cells were cryopreserved for potential future use. After two weeks of culture, single-cell-derived colonies were manually picked, transferred individually to Geltrex-coated 96-well plates, and expanded. Once sufficient cell numbers were obtained, clones were cryopreserved, and DNA was isolated from a subset for further screening.

Verification of the absence of Sendai Virus

To confirm the absence of Sendai virus (SeV) components from the hiPSC lines, we conducted PCR analysis targeting viral sequences. Total RNA was extracted from hiPSC cultures using the Total RNA Mini Kit (A&A Biotechnology) according to the manufacturer's protocol. Reverse transcription was performed using 1 µg of total RNA as template with the High-Capacity cDNA Reverse Transcription Kit (Thermo Fisher Scientific) in a ProFlex PCR System (Thermo Fisher Scientific). Subsequent PCR amplification utilized 30 ng of cDNA template with Sendai virus-specific primers (forward: 5'-GGATCACTAGGTGATATCGAGC-3'; reverse: 5'-ACCAGACAAGAGTTTAAGAGATATGTATC-3') and KAPA2G Fast Genotyping Mix (Roche). The PCR reaction was performed under the following conditions: initial denaturation at 95 °C for 3 minutes, followed by 30 cycles of 95 °C for 20 seconds, 60 °C for 15 seconds, and 72 °C for 15 seconds, with a final extension at 72 °C for 2 minutes. PCR products were analyzed by electrophoresis on 2 % agarose gels in TAE buffer at 70 V for 45 minutes, stained with ethidium bromide, and visualized under UV transillumination.

Verification of Mycoplasma spp. contamination

To detect potential Mycoplasma contamination in cell cultures, we performed PCR analysis targeting conserved Mycoplasma-specific sequences. Total DNA was extracted from cultured cells using the Genomic Mini Kit (A&A Biotechnology) according to the manufacturer's protocol. PCR amplification was conducted using 50 ng of genomic DNA template with mycoplasma-specific primers (forward: 5'-ACTCCTACGGGAGGCAGCAG-3'; reverse: 5'-TGCACCATCTGTCACTCTGTTAACCTC-3') and KAPA2G Fast Genotyping Mix (Roche). The PCR reaction was performed under the following conditions: initial denaturation at 95 °C for 3 minutes, followed by 30 cycles of 95 °C for 20 seconds, 67 °C for 15 seconds, and 72 °C for 15 seconds, with a final extension at 72 °C for 2 minutes. PCR products were analyzed by electrophoresis on 2 % agarose gels in TAE buffer at 70 V for 45 minutes, stained with ethidium bromide, and visualized under UV transillumination.

Detection of HIV integration

To detect potential HIV genome integration in hiPSC lines, we performed PCR-based analysis targeting HIV-specific sequences. Total DNA was extracted from cultured cells using the Genomic Mini Kit (A&A Biotechnology) according to the manufacturer's protocol. PCR amplification was conducted using 50 ng of genomic DNA template with two HIV-specific primer pairs targeting the TAR region (HIV target 1; forward: 5'-GTCTCTCTGGTTAGACCAG-3'; reverse: 5'-TGGGTTCCCTAGTTAGCC-3') and RRE region (HIV target 2; forward: 5'-AGTGGTGCAGAGAGAAAAAGAGC-3'; reverse: 5'-TGGGTTCCCTAGTTAGCC-3') using KAPA2G Fast Genotyping Mix (Roche). The PCR reaction was performed under the following conditions: initial denaturation at 95 °C for 3 minutes, followed by 30 cycles of 95 °C for 20 seconds, 60 °C for 15 seconds, and 72 °C for 15 seconds, with a final extension at 72 °C for 2 minutes. PCR products were analyzed by electrophoresis on 2 % agarose gels in TAE buffer at 70 V for 45 minutes, stained with ethidium bromide, and visualized under UV transillumination.

The HIV positive control and primers targeting HIV were kindly provided by dr hab. Anna Kula-Păcurar.

Karyotype genetic test

hiPSCs were cultured in vitro under standard conditions. Following karyotype maintenance procedures, chromosome preparations were subjected to Wright staining. Metaphase spreads were analyzed using the GenASIs imaging software. All analyses were performed by certified cytogenetic specialists at Gyncentrum Kraków, Poland.

Supplementary tables:

Supplementary Table 1. List of used primers

Target	Purpose	Primer sequence (5'→3')	Tm (°C)
HNF1A	Surveyor assay and Sanger sequencing	F: CATGGTTTCTAAACTGAGCCAGCT<br>R: CAGCAACTAGGCTAAAGGCTGAAG	62
GSE1	Off-target sgRNA activity	F: GGAGTTGAGTCGGTTTGGCT<br>R: GCTCCTGAATCTACCTGGGC	60
HNF1A	Real time PCR	F: GTGGCGTGGCGAAGAT<br>R: CTTCATGGGAGTGCCCTTGTT	60
EEF2	Real time PCR	F: TCAGCACACTGGCATAGAGG<br>R: GACATCACCAAGGGTGTGCA	60

Supplementary Table 2. PCR KAPPA program

Stage	Temperature (°C)	Time (s)	Cycles
**Initial Denaturation**	95	180	1
**Denaturation**	95	20	30
**Annealing**	Depends on the primer Tm	15	
**Extension**	72	45	
**Final Extension**	72	120	1

Supplementary Table 3. The list of selected gRNA candidates. The order was set by the increasing predicted off-target activity.

Name	gRNA sequence 5'→3' (PAM)
gRNA-1	AGGACGAGACGGACGACGAT (GGG)
gRNA-2	GACGAGACGGACGACGATGG (GGG)
gRNA-3	TCGGGGCTCCGAGGACGAGA (CGG)

Supplementary Table 4. List of used antibodies and dyes

Antibody/Dye	Type	Dilution/ concentration	Catalog number	Company
**Rabbit anti-Oct 3/4**	Primary ab.	1:200	sc-5279	Santa Cruz Biotechnology
**Mouse anti-Nanog**	Primary ab.	1:100	sc-33759	Santa Cruz Biotechnology
**Mouse anti-TRA-1-60**	Primary ab.	1:100	SCR001 (90232)	Merck
**Mouse anti-TRA-1-81**	Primary ab.	1:100	SCR001 (90233)	Merck
**Mouse GATA-4**	Primary ab.	1:200	sc-25310	Santa Cruz Biotechnology
**Rabbit Vimentin**	Primary ab.	1:200	MA5-35320	Invitrogen
**Mouse Tubulin-β3**	Primary ab.	1:200	801213	BioLegend
**Rabbit HNF1A**	Primary ab.	1:1000	22426-1-AP	Proteintech
**Rabbit GAPDH**	Primary ab.	1: 20000	14C10	Cell Signaling
**Donkey anti-rabbit Alexa-Fluor 488**	Secondary ab.	1:500	A21206	Thermo Fisher Scientific
**Donkey anti-mouse Alexa-Fluor 568**	Secondary ab.	1:500	A10037	Thermo Fisher Scientific
**Goat anti-rabbit-HRP**	Secondary ab.	1: 10000	G-21234	Thermo Fisher Scientific
**DAPI**	DNA dye	0.2 μg/ml	D9542	Sigma-Aldrich
