# Supplementary material for: CRISPR/Cas9-based repair of a heterozygous HNF1A mutation in patient-derived hiPSCs
Source: Hum Genet. 2026 Aug 12;145(1):69. doi: 10.1007/s00439-026-02863-0 (PMC13469492; doi:10.1007/s00439-026-02863-0)
Supplement: Supplementary file 2 [file 439_2026_2863_MOESM2_ESM.pdf]

# CRISPR/Cas9-based repair of a heterozygous HNF1A mutation in patient-derived hiPSCs

*Dawid Skoczek[1,2], Jerzy Hohendorff[3], Maciej T. Malecki[3], Alicia Roig-Merino[4], Rasmus O. Bak[5], Neli Kachamakova-Trojanowska[1*]*

Supplementary Figures

S1-S5

**A**

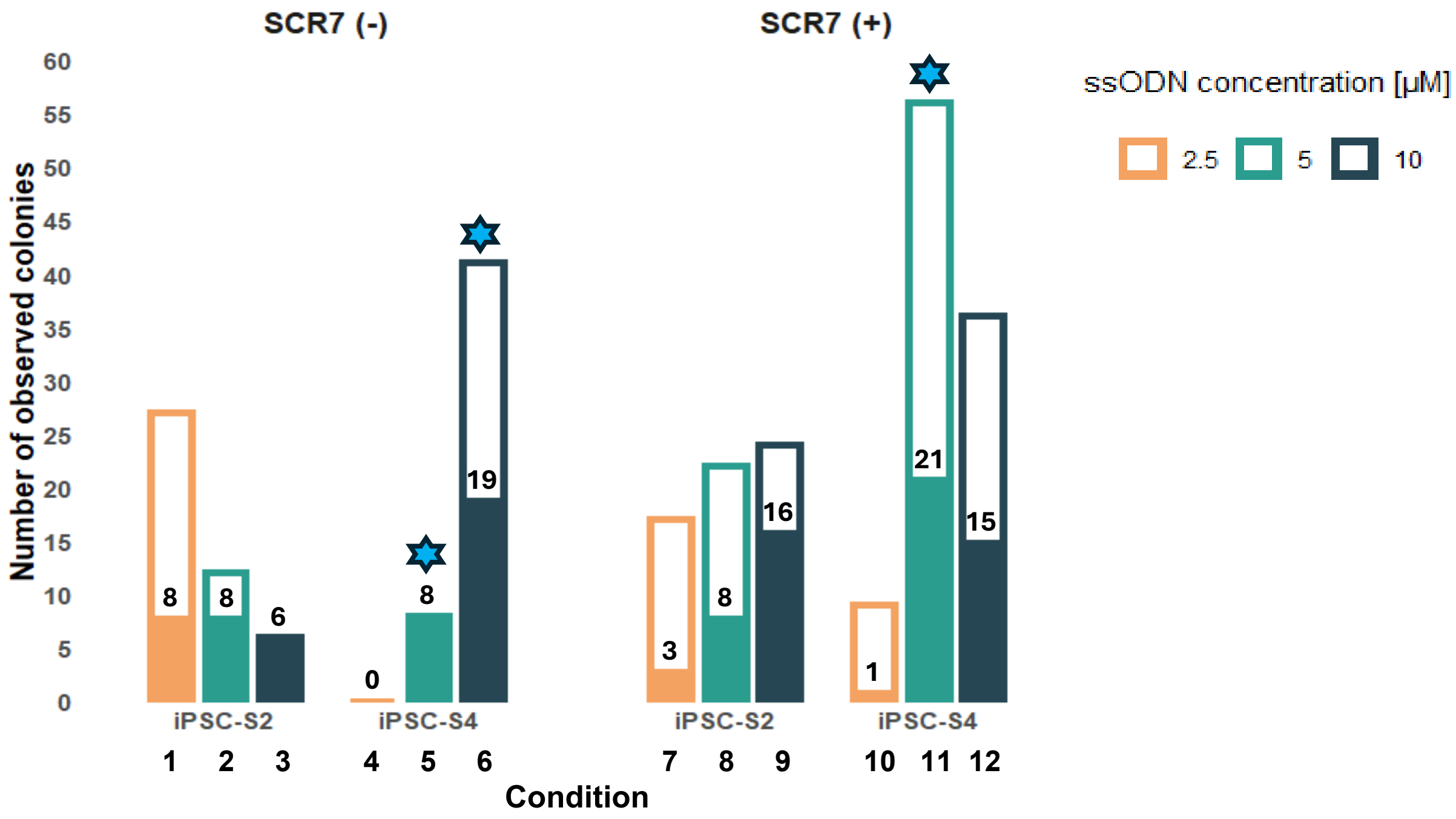


**B**

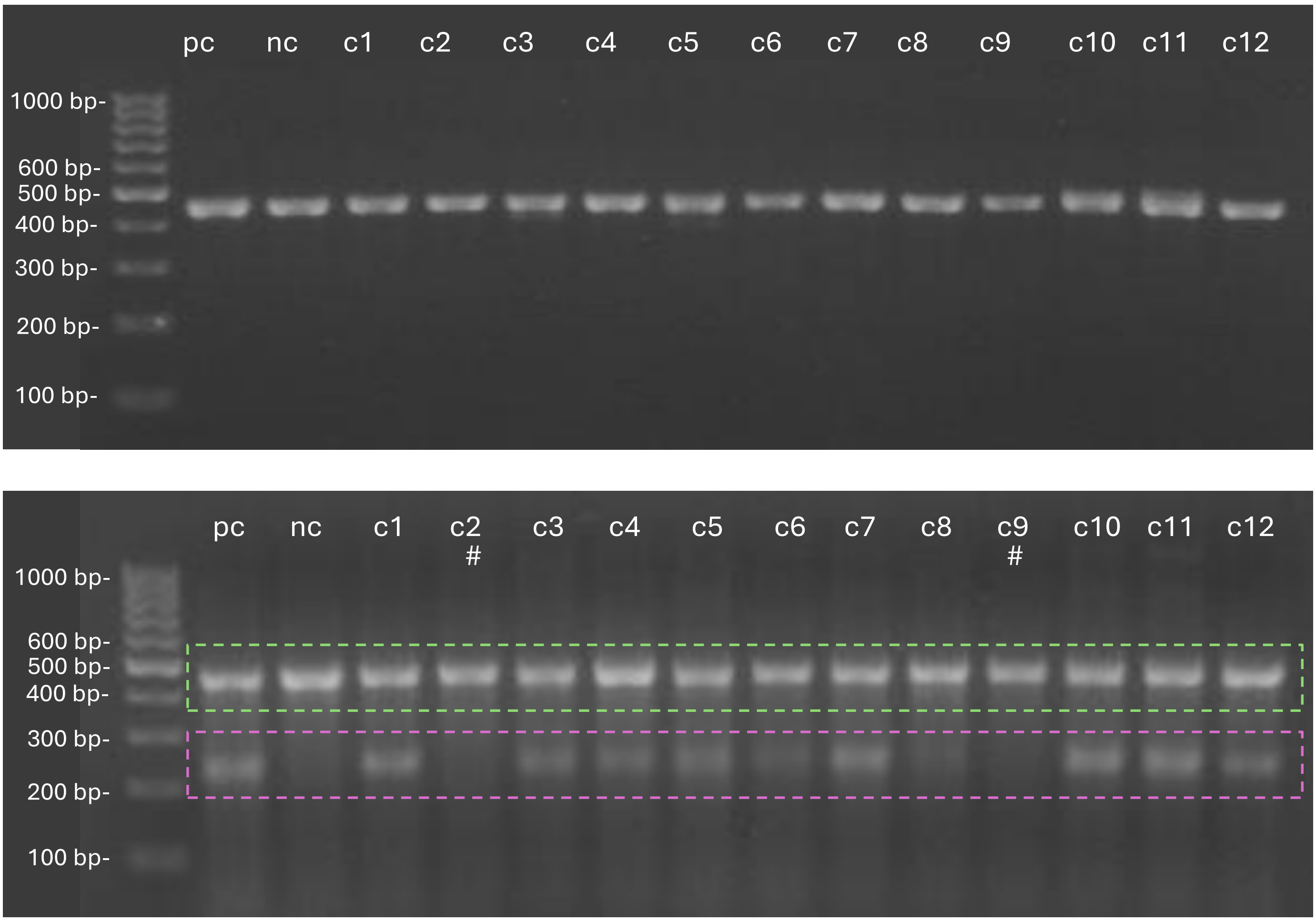


**Fig. S1** (A) Summary of the number of colonies observed on 10 cm culture dishes after single-cell seeding, following nucleofection and plating. The colored area indicates colonies screened using the Surveyor assay. Differences between observed and screened colonies result from unsuccessful transfers to 96-well plates and the exclusion of those that lost pluripotency or deviated from the hiPSC-specific phenotype, rendering them unsuitable for further processing. Conditions in which the mutation was successfully corrected are indicated by a blue star. Colonies from condition 11, obtained and screened after melting, are not included in the graph. (B) Representative results of the Surveyor assay for 12 tested clones (c). The upper panel depicts the electrophoretic separation of the specific PCR-amplified *HNF1A* fragment of the mutation site. The lower panel shows the effect of Surveyor nuclease digestion. Specifically, the appearance of a band at approximately 250 bp (highlighted with a purple frame) indicates the presence of heteroduplexes due to mismatches that were cleaved by the nuclease, thereby indicating the presence of mutations. Clones marked with a hash (#) were considered promising and selected for Sanger sequencing. The positive control (pc) represents the original patient-specific line, while the negative control (nc) corresponds to the wild-type HNF1A line

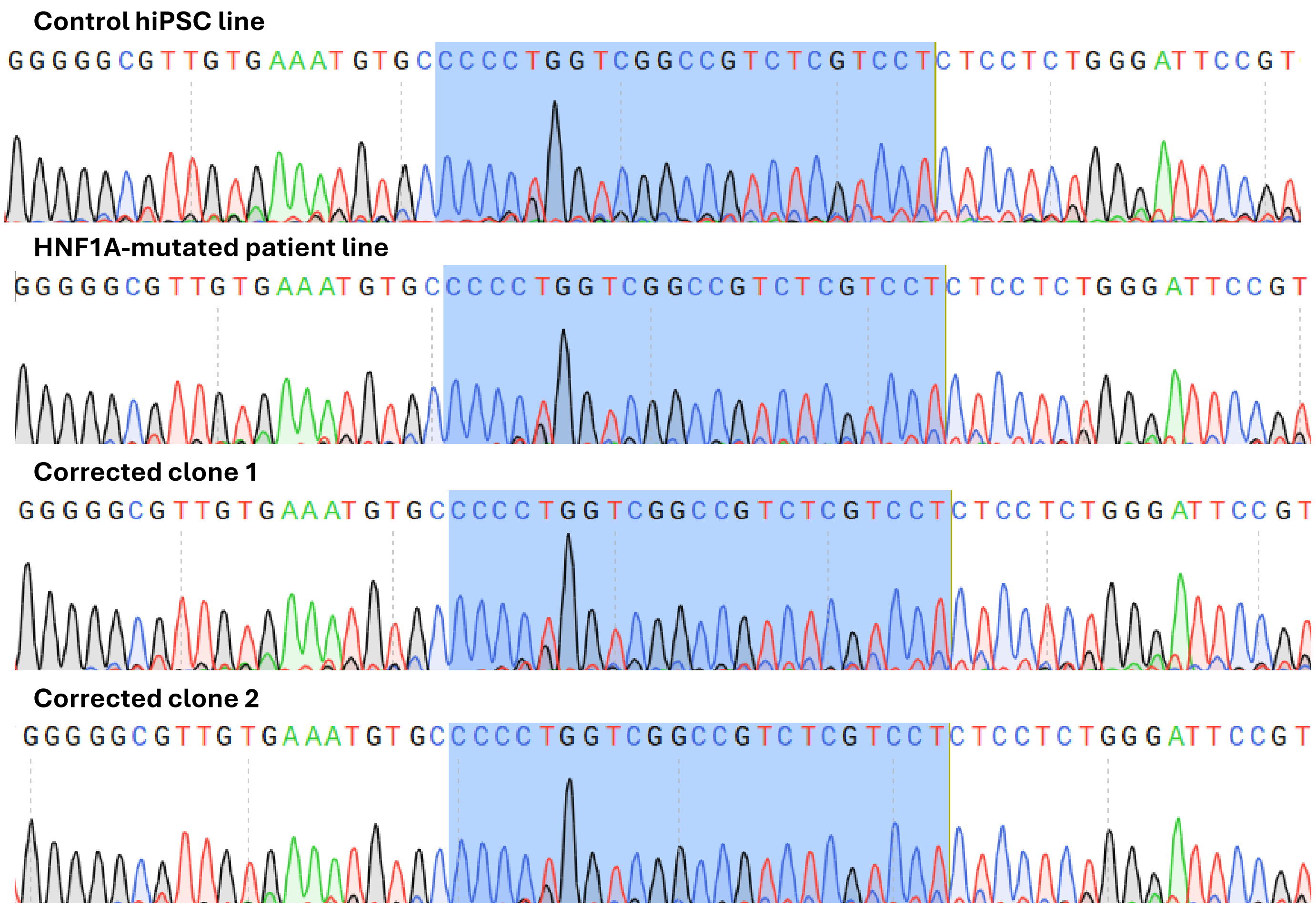


**Fig. S2.** Sanger sequencing results of the off-target analysis in the hiPSC control line, HNF1A-mutated patient line, and corrected clones for the most likely off-target site located in the intronic region of the GSE1 gene. The potential off-target site is highlighted in blue. No indels were detected following CRISPR editing.

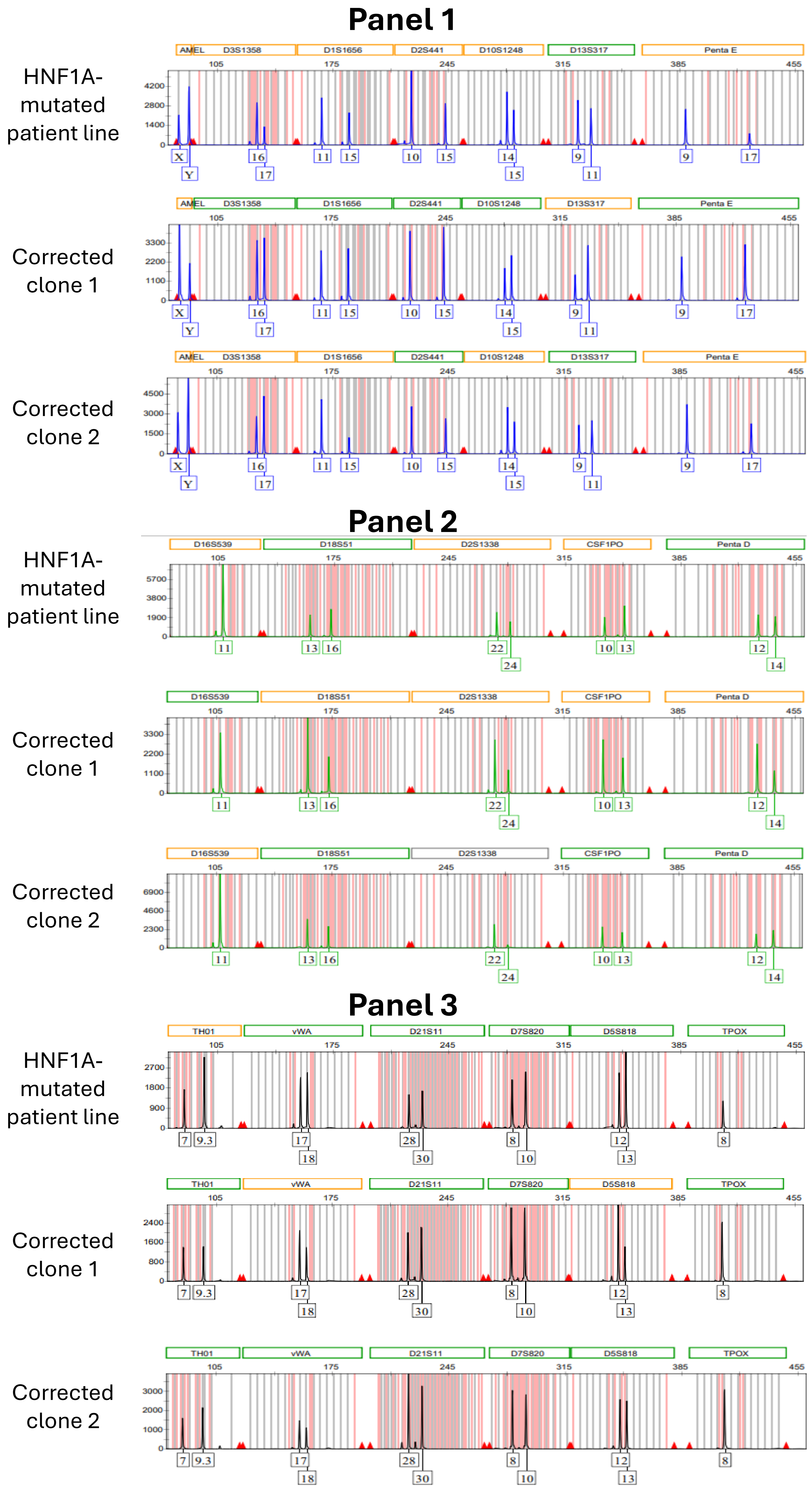


**Fig. S3**. Representative electropherograms of short tandem repeat (STR) analysis confirming genetic identity between donor cells and gene-corrected clones.

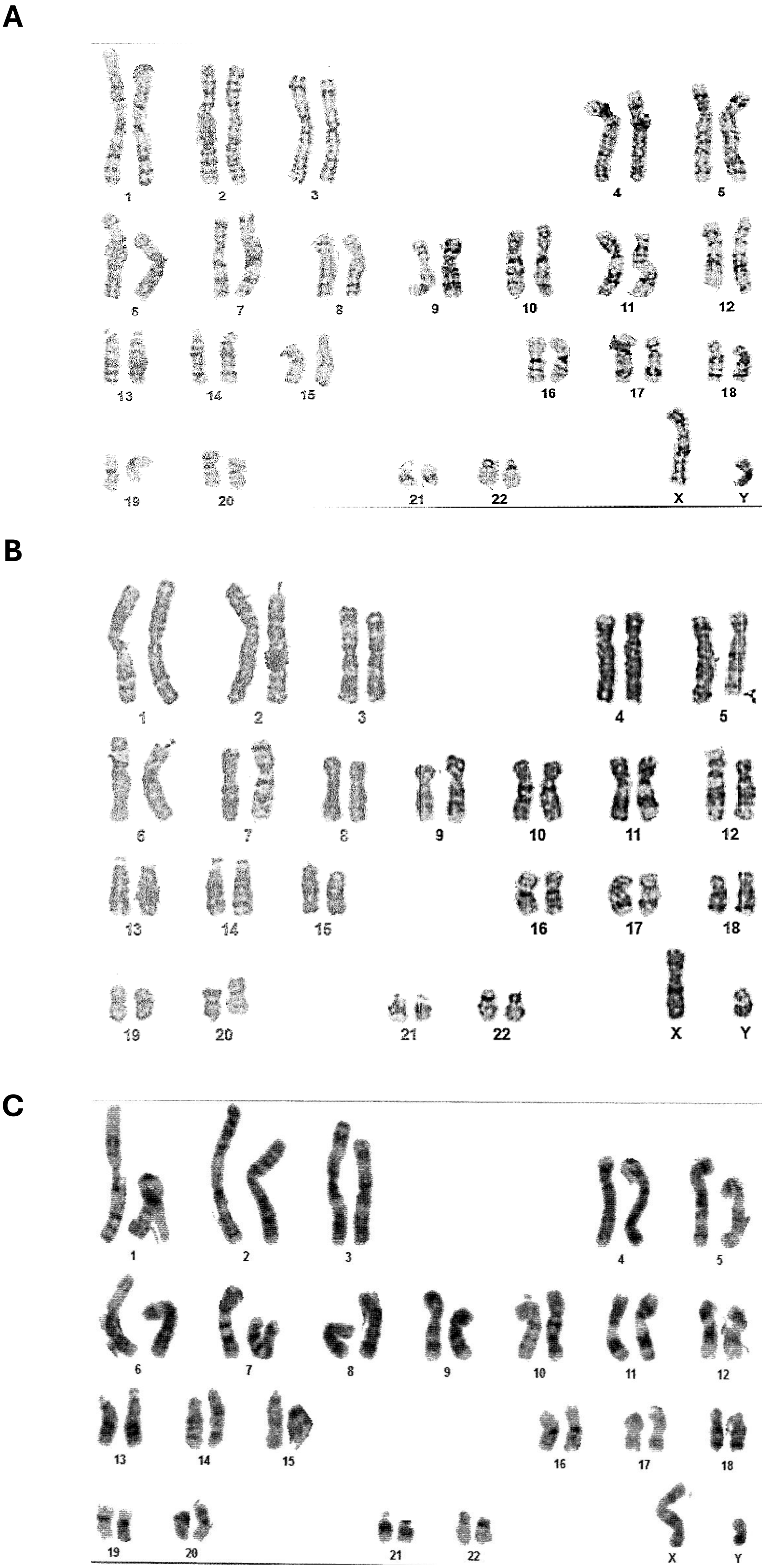


**Fig. S4**. Representative karyotype results of the corrected clones (A and B) and the patient-specific line (C). All karyotypes demonstrate a correct chromosome content and structure.

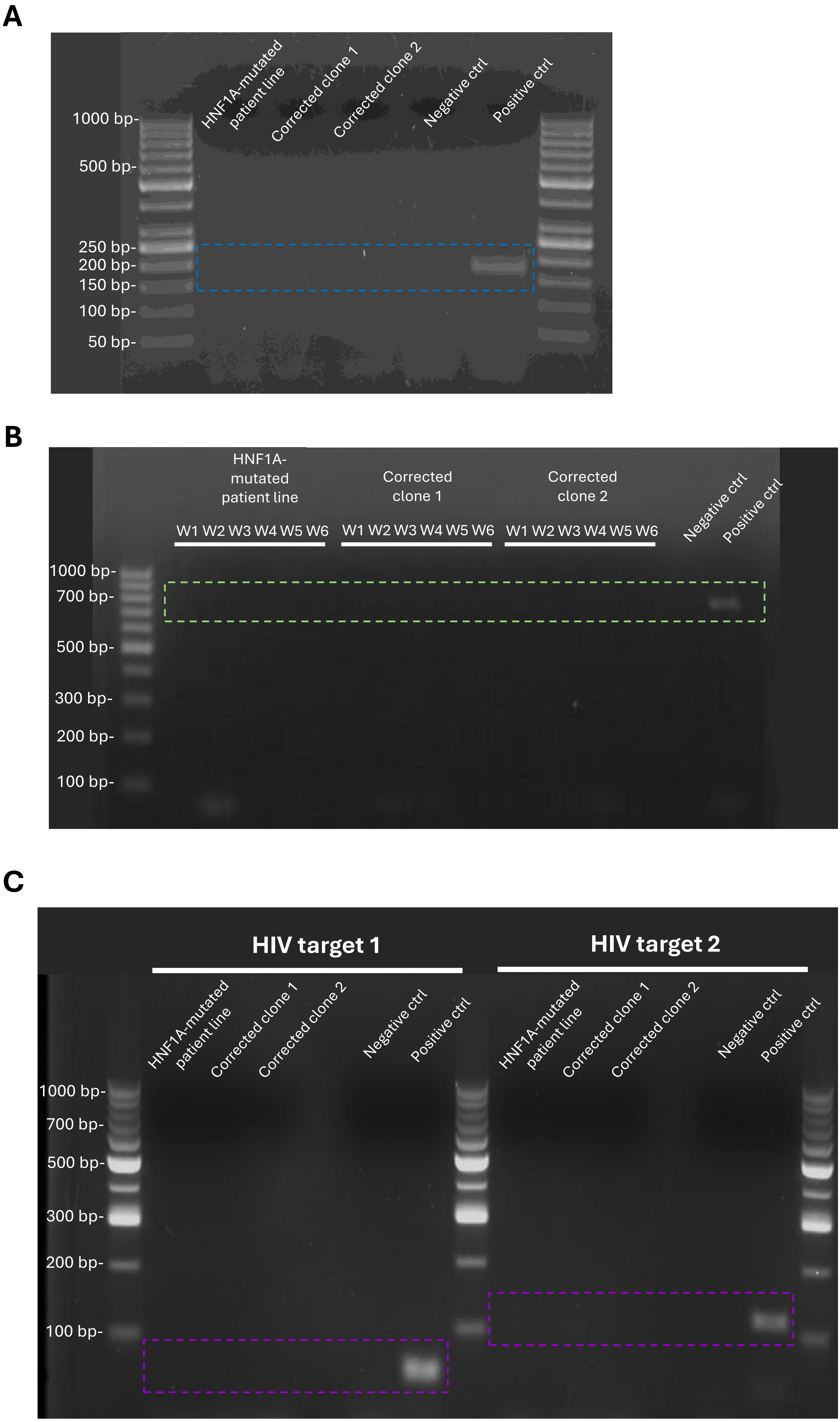


**Fig. S5**. (A) PCR analysis confirming the absence of the reprogramming vector (Sendai virus) in hiPSC HNF1A-mutated patient-specific line and corrected clones. The positive control was obtained from PBMCs on day three of reprogramming. (B) PCR-based detection of Mycoplasma spp. contamination in hiPSC lines conducted over a six-week (W) period. (C) PCR-based detection of HIV genome integration in hiPSC lines (HIV target 1 – Trans-Activation Response element; HIV target 2 – Rev Response Element).
